# A Fluorescence-Based Acetylcholinesterase Inhibition Assay for Bioactivity-Guided Identification of Neuroactive Protoberberine Alkaloids from *Berberis julianae* C.K. Schneid.

**DOI:** 10.3390/molecules31183182

**Published:** 2026-09-10

**Authors:** Maryna Koval, Magdalena Lasota, Aleksandra Barańska, Bartosz Skóra, Myroslav Shevera, Tetiana Dvirna, Katarzyna Gaweł-Bęben, Wirginia Kukula-Koch

**Affiliations:** 1 Department of Pharmacognosy with Medicinal Plants Garden, Medical University of Lublin, 1, Chodzki Str., 20-093 Lublin, Poland; marinchik.koval@gmail.com; 2 Doctoral School, Medical University of Lublin, 7 Chodzki Str., 20-093 Lublin, Poland; 3 Department of Cosmetology, Faculty of Medicine, University of Information Technology and Management in Rzeszów, 2, Sucharskiego Str., 35-225 Rzeszów, Poland; mlasota@wsiz.edu.pl (M.L.); abaranska@wsiz.edu.pl (A.B.); kagawel@wsiz.edu.pl (K.G.-B.); 4 Department of Biotechnology and Cell Biology, Faculty of Medicine, University of Information Technology and Management in Rzeszów, 2, Sucharskiego Str., 35-225 Rzeszów, Poland; bskora@wsiz.edu.pl; 5 M.G. Kholodny Institute of Botany, National Academy of Sciences of Ukraine, 2, Tereshchenkivska Str., 010601 Kyiv, Ukraine; shevera.myroslav@ukr.net (M.S.); dvirna_t@ukr.net (T.D.); 6 Department of Pharmacognosy and Botany, Bogomolets National Medical University, 13, T. Shevchenko Blvd., 010601 Kyiv, Ukraine

**Keywords:** acetylcholinesterase inhibitors, bioactivity assay, fluorimetric tests, in vitro studies in cells, isoquinoline alkaloids, barberry, *Berberis* spp.

## Abstract

Background/Objectives: The identification of acetylcholinesterase (AChE) inhibitors from complex natural matrices remains challenging due to the limitations of conventional colorimetric assays. This study aimed to develop a fluorescence-based AChE inhibition assay (FAIA) suitable for screening plant extracts, while simultaneously characterizing the metabolite profile of *Berberis julianae* fruits, identifying bioactive alkaloid-enriched fractions, and evaluating their cytotoxicity in neuronal cell models. Methods: Methanolic extracts of *B. julianae* fruits were profiled by HPLC-ESI-QTOF-MS/MS and fractionated using centrifugal partition chromatography (CPC). The developed FAIA, based on 4-methylumbelliferyl acetate as a fluorogenic substrate, was optimized and validated using berberine as a reference inhibitor. CPC fractions and selected protoberberine alkaloids were screened for AChE-inhibitory activity, while cytotoxicity was assessed in differentiated and undifferentiated SH-SY5Y cells using the resazurin assay. Results: Twenty-five metabolites, including eight isoquinoline alkaloids, were tentatively identified in the fruit extract. The optimized FAIA enabled reliable evaluation of AChE inhibition without the limitations associated with chromogenic assays. Among the CPC fractions, fraction 7 exhibited the strongest inhibitory activity. LC-MS analysis revealed that this fraction was enriched in protoberberine alkaloids, including berberine, palmatine, jatrorrhizine, magnocurarine, and demethyleneberberine. Cytotoxicity studies demonstrated concentration-dependent effects of both the isolated fractions and the individual alkaloids, with differentiated and undifferentiated SH-SY5Y cells exhibiting distinct sensitivity profiles. At 200 µg/mL, cell viability ranged from approximately 20–40% in undifferentiated SH-SY5Y cells and from approximately 25–45% in differentiated SH-SY5Y cells, depending on the CPC fraction. Among the tested protoberberine alkaloids, demethyleneberberine exhibited the lowest cytotoxic effect, maintaining the highest viability of differentiated SH-SY5Y cells, followed by jatrorrhizine and berberine. Conclusions: The proposed FAIA represents a sensitive and practical approach for screening AChE inhibitors in complex plant matrices. Combined with metabolomic profiling and CPC-based bioassay-guided fractionation, it enabled the identification of alkaloid-rich fractions of *B. julianae* with pronounced anti-AChE activity. These findings support the applicability of the developed workflow for natural-product-based drug discovery targeting neurodegenerative disorders.

## 1. Introduction

Acetylcholinesterase inhibitors (AChE inhibitors) are compounds that block the activity of the enzyme acetylcholinesterase (AChE), thereby increasing the levels of acetylcholine (ACh) in synaptic clefts and enhancing cholinergic transmission. They are currently the main class of drugs used for the treatment of Alzheimer’s disease (AD). The exact cause of Alzheimer’s disease is not fully understood, as it involves a combination of genetic, environmental, and lifestyle factors. One of the existing theories of AD is an impairment of cholinergic function. People with AD are deficient in ACh in comparison with healthy people. ACh is an important neurotransmitter produced by cholinergic neurons and is closely related to memory and learning ability [1]. Even if there is no cure for AD, some AChE inhibitors can mitigate the progression of the disease and manage some of the symptoms. Donepezil, rivastigmine, and galantamine are used in modern clinical practice as first-line drugs in mild and advanced AD. Despite their clinical benefits, currently available AChE inhibitors are associated with adverse effects and provide only symptomatic relief, with limited long-term efficacy. Therefore, the discovery of new AChE inhibitors remains an important goal in the development of improved therapeutic strategies for Alzheimer’s disease [2,3].

The search for promising candidates that effectively inhibit AChE can be conducted using various assays, ranging from in vitro to in vivo methods. Among the commonly used assays, Ellman’s test is widely used to determine AChE activity. This traditional method, used for decades, relies on the hydrolysis of acetylthiocholine iodide by AChE to produce thiocholine, which reacts with 5,5′-dithiobis(2-nitrobenzoic acid) (DTNB), forming a yellow-colored product (5-thio-2-nitrobenzoic acid). In this method, AChE is incubated with the inhibitor, and acetylcholine iodide is added as the substrate. Even though the Ellman assay is simple, rapid, sensitive, and relatively low-cost, it has several disadvantages. The substances under study need to react with DTNB. Also, because it is a colorimetric assay, compounds that absorb UV at 412 nm can yield false-positive results. In the scientific literature, we also find several modifications of this original test, e.g., that use a different substrate, like ACh, forming choline as the final product [2]; naphthyl acetate, leading to 1-naphthol production, which reacts with fast blue salt and forms a purple-colored azo dye [4]; and others.

The identification of AChE inhibitors can be performed using other techniques, e.g., radiometric assays with radiolabeled acetylcholine (e.g., [14C]-acetylcholine) to measure AChE activity by detecting radiolabeled products. In this highly reproducible and sensitive assay, a radiolabeled ACh is used as a substrate, and the reaction products are separated and quantified using scintillation counting. Even if highly accurate, this method poses a disposal problem due to the radioactive waste produced in the experiment [5]. Also, electrochemical techniques can be used to detect potential AChE inhibitors. The assays use electrochemical sensors (conductometric, potentiometric, or amperometric) to detect the products of AChE activity, with ACh as the substrate [6].

Fluometric assays—the last of the comprehensively described techniques in the scientific literature—were of interest to the authors of this manuscript. The approach uses a fluorogenic substrate, such as 4-methylumbelliferyl acetate, coupled with a fluorogenic dye to detect AChE activity. In the assay, substrate hydrolysis generates a fluorescent product that is quantified using specific excitation and emission wavelengths. Among the main advantages of the method are its high sensitivity and its ability to detect colored compounds that are difficult to detect with colorimetric methods. Although numerous assays are available for determining acetylcholinesterase inhibition, many have limitations when applied to complex matrices such as plant extracts due to interference from endogenous compounds. Therefore, there is still a need to develop new assays that are more selective and less influenced by sample properties, thereby providing more reliable and unbiased results.

In this study, a fluorimetric-based approach was introduced to evaluate the AChE-inhibitory potential of natural products. For this purpose, isoquinoline alkaloids present in the extract of *Berberis julianae* were selected to assess their bioactivity, but also to constitute a sample for the evaluation of the activity of a new assay.

*Berberis* is a large genus of deciduous and evergreen shrubs, with about 600 species, characterized by yellow wood and flowers, and common in temperate and subtropical regions of the world. It has strong potential for a wide range of applications in the food and pharmaceutical industries due to its nutritional and therapeutic properties, including antimicrobial, antiemetic, antipyretic, antioxidant, anti-inflammatory, antidiabetic, antiarrhythmic, antihypertensive, sedative, anticholinergic, cholagogic, antileishmanial, and antimalarial actions [7]. Owing to its bactericidal and wound-healing properties, together with its high vitamin content, barberry is a valuable cosmetic plant. It is widely incorporated into skin care products intended for sun-damaged or dry skin with signs of aging [8].

One of the most important alkaloids of *Berberis* spp. is berberine (BER), an isoquinoline derivative with extensively researched pharmacological potential. This alkaloid, present in various plant parts, such as bark, roots, and leaves, is often used for its antibacterial, antiprotozoal, antidiarrheal, antidiabetic, antihypertensive, antidepressant, and anti-inflammatory properties [9]. In addition, numerous scientific works confirm the ability of BER to inhibit acetylcholinesterase AChE [10,11]. BER is considered a potential compound that could be used as an AChE inhibitor in the treatment of AD [12].

The alkaloid composition of barberry may vary depending on the species, geographical origin, harvesting period, or processing method. While BER is a common and significant alkaloid found across many species, other compounds contribute to each species’ unique profile. One of the many species belonging to the Berberidaceae family is *Berberis julianae* C.K. Schneid. (wintergreen barberry or Chinese barberry), a hardy evergreen shrub native to central China. It is also widely cultivated as an ornamental plant in temperate regions. Scientific studies on *Berberis julianae* are very limited; therefore, this species has attracted increasing interest, particularly regarding its chemical composition and the potential of its constituents as AChE inhibitors. In this study, the phytochemical profile of *Berberis julianae* fruits is reported, along with a bioassay-guided fractionation using centrifugal partition chromatography (CPC) to identify individual constituents responsible for the significant inhibitory potential of the total extract. Another aim of this study was to develop a fluorimetric AChE inhibition assay to screen potential drug candidates for further investigation as treatments for Alzheimer’s disease.

Since the development of neuroprotective agents requires not only biological activity but also an acceptable safety profile, the cytotoxicity of the selected CPC fractions and protoberberine alkaloids was evaluated in SH-SY5Y cells. Assessing cell viability is essential to determine whether adverse effects accompany the observed AChE-inhibitory activity on neuronal cells and to identify concentrations that retain biological activity without inducing excessive cytotoxicity. Human SH-SY5Y neuroblastoma cells are among the most widely used in vitro models in neuroscience research, particularly for studies of neurotoxicity, neurodegeneration, and neuroprotective agents [12,13,14]. Upon differentiation, SH-SY5Y cells acquire morphological and biochemical characteristics of mature neurons, making them a more physiologically relevant neuronal model [15]. Since differentiated and undifferentiated SH-SY5Y cells may respond differently to biologically active compounds, both cell models were employed in the present study to comprehensively evaluate the cytotoxicity of the selected CPC fractions and protoberberine alkaloids.

## 2. Results

The fluorescence-based acetylcholinesterase inhibition assay (FAIA) developed in this study was designed as a rapid, straightforward tool for screening natural products for potential anti-AChE activity. To demonstrate its applicability, the assay was applied to fractions from *Berberis julianae* fruits, a rich source of isoquinoline alkaloids, a class of natural compounds previously reported to exhibit acetylcholinesterase-inhibitory activity. Following assay validation, the optimized protocol enabled efficient bioactivity-guided screening of CPC fractions, thereby identifying alkaloid-enriched fractions with the highest inhibitory potential. The simplicity of the fluorescence readout, together with the absence of chromogenic interference commonly encountered in colorimetric methods, makes FAIA a practical alternative for evaluating complex plant matrices.

### 2.1. FAIA Action Principle

The basis for the FAIA was the fluorescent Gainche probe [13]. In their experiment, the authors modified the method that used 1-naphthyl acetate and fast blue salt (FBS) as inhibitor detectors, replacing 1-naphthyl acetate with 4-methylumbelliferone acetate. 4-MU acetate is commonly used as a fluorescent substrate in biochemical assays. 4-MUA is non-fluorescent or weakly fluorescent in its native state, but when hydrolyzed by esterases, it exhibits strong blue fluorescence. The excitation wavelength of coumarin is around 360 nm, and the emission is 450 nm.

**Figure 1:**
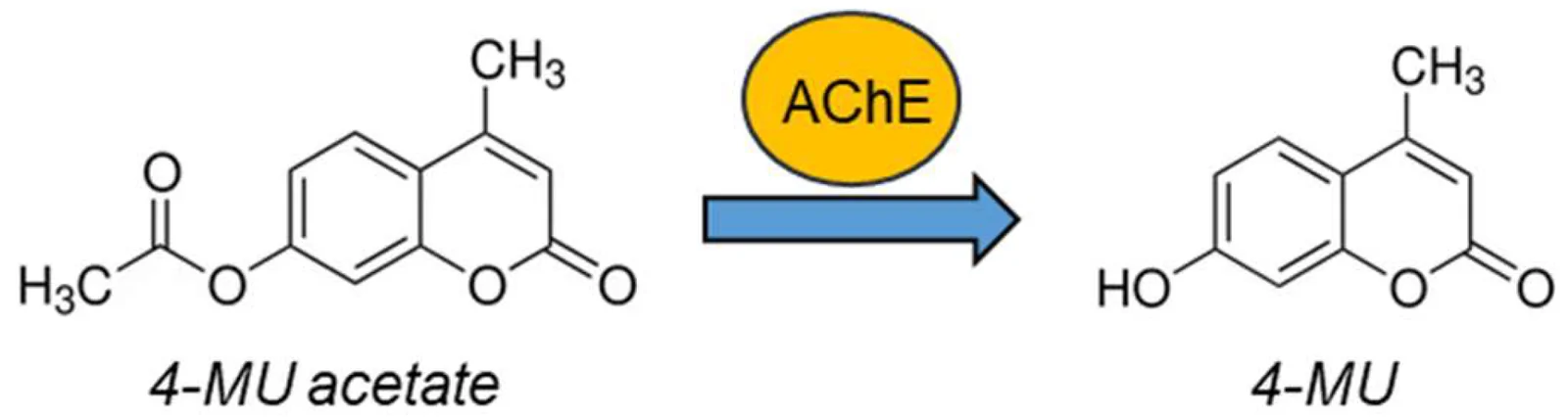
Enzymatic hydrolysis of 4-methylumbelliferyl acetate (4-MU acetate) to fluorescent 4-methylumbelliferone (4-MU).

The Gainche assay was performed on the HPTLC plates. HPTLC plates were derivatized with 4-MU acetate, then dried and observed at 366 nm. Non-fluorescent spots on a fluorescent background showed the presence of inhibitors. A similar phenomenon can be observed in FAIA: weakly fluorescing wells after applying probes showed a high percentage of inhibitory activity. Instead, the blank with a solvent (DMSO), which shows no activity relative to AChE, fluoresces brightly. The reason for this lies in the test’s principle of action, which is based on the interaction between 4-methylumbelliferone acetate (4-MUA) and the enzyme (Figure 1). Coumarin in the acetate does not exhibit fluorescence; fluorescence occurs only after hydrolysis by AChE. Thus, when the AChE inhibitor binds the enzyme, less AChE can react with 4-MU acetate (non-fluorescent), and the substance cannot change to its fluorescent state, 4-MU.

### 2.2. FAIA Optimization

First, the general protocol for the assay was optimized. The volumes of the tested samples were checked in the range of 5–20 µL, the concentration range was adjusted, and the concentration of the 4-MUA was set. Before reaching the final conditions of the experiment, the plate was tested in absorbance mode to select the appropriate concentrations of the sample, enzyme, substrate, and standard at wavelengths of 405 and 320 nm. The best results were obtained when mixing 10 µL of a sample with 180 µL of enzyme (3 U/mL) and, after 25 min of incubation at 37 °C, with 10 µL of a 4-MUA solution at 1.5 mg/mL dissolved in 50% ethanol. The protocol was a modification of the original Elmann’s test [14].

The selected assay conditions provided a stable fluorescence signal with a favorable signal-to-background ratio and reproducible measurements across replicate wells. The optimized protocol was subsequently applied throughout the study without further modification, ensuring methodological consistency during the screening of plant extracts and CPC fractions.

**Figure 2:**
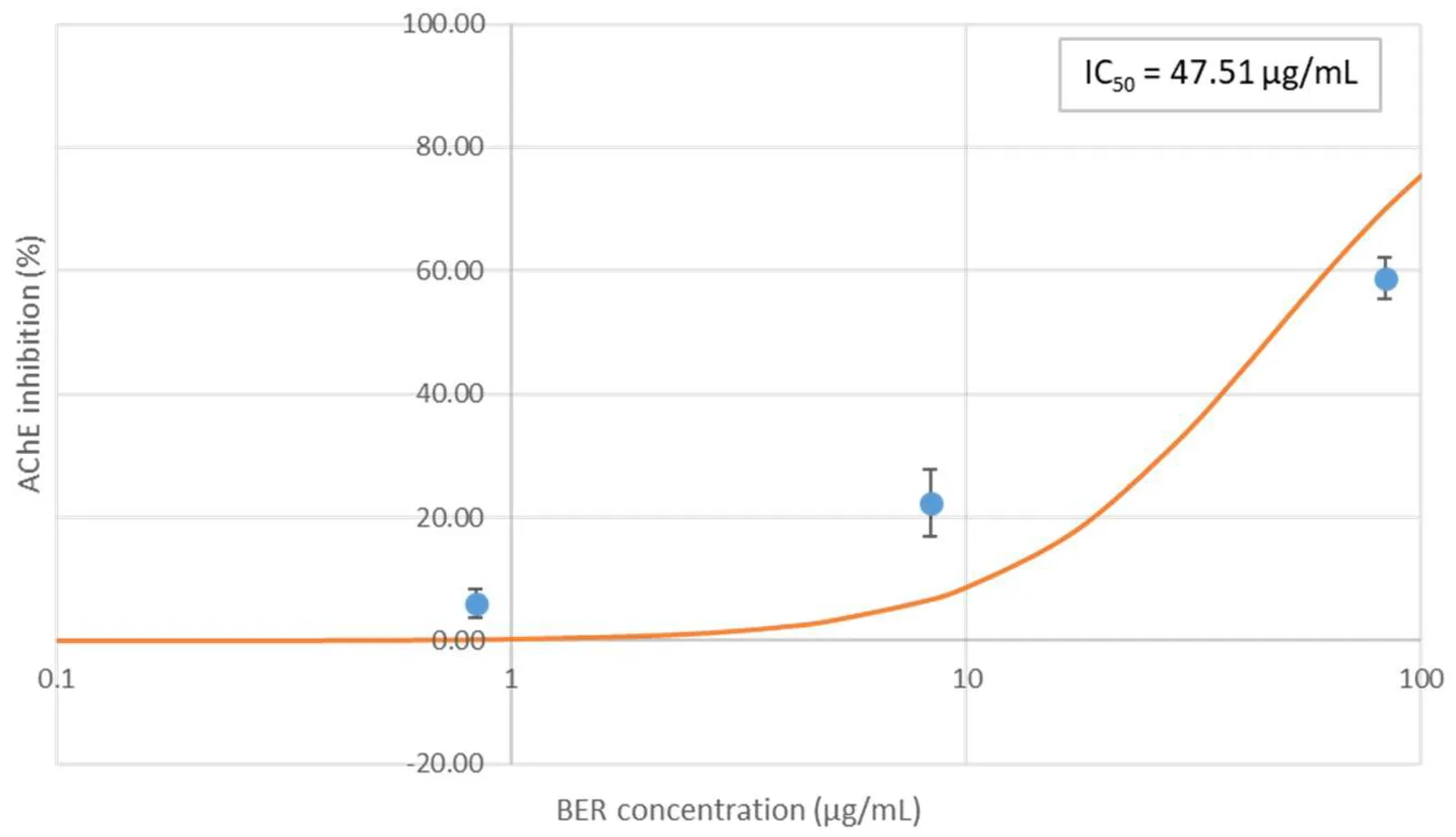
Concentration-dependent inhibitory effect of BER on AChE activity and determination of the IC_50_ value. Data are presented as mean ± SD (*n* = 3). The fitted concentration–response curve was used to determine an IC_50_ value of 47.51 µg/mL in the well (approximately 141 µM).

Berberine was selected as the reference inhibitor in this assay due to its well-documented AChE-inhibitory activity; moreover, it yielded consistent, reproducible results during method optimization and preliminary experiments. The inhibitory activities of berberine were compared by determining its IC_50_ values (see Figure 2). For this purpose, a preliminary concentration–response study was performed at four concentrations: 1, 0.1, 0.01, and 0.001 mg/mL. As a result, berberine exhibited a concentration-dependent inhibitory effect, reaching approximately 50% inhibition at 0.84 mg/mL, thereby enabling estimation of its IC_50_ value. The concentration-dependent response observed for berberine confirmed FAIA’s ability to reliably detect these inhibitors with concentration-dependent varying inhibitory potencies and with a reproducible inhibition profile.

The optimization experiments demonstrated that the proposed FAIA provides reproducible measurements of AChE inhibition under the selected experimental conditions. The assay detected concentration-dependent inhibition by the reference compound. Furthermore, the intrinsic fluorescence of the tested fractions and selected individual alkaloid standards was evaluated using sample-specific fluorescence blanks, thereby accounting for background fluorescence in the data analysis. Taken together, these results support the applicability of the optimized FAIA for the preliminary bioactivity-guided evaluation of natural products and fractionated extracts. Further systematic validation is required to characterize the assay’s analytical performance fully. To exclude potential interference with the fluorescence measurements, the intrinsic fluorescence of berberine was evaluated under the assay conditions. Background fluorescence was measured for BER and later for other samples at the tested concentrations in the absence of enzyme and substrate (see Figure S1 in Supplementary Materials).

This control experiment was important because many plant-derived alkaloids and complex plant fractions possess intrinsic optical properties that may interfere with fluorescence-based measurements. Sample-specific fluorescence blanks were therefore evaluated for the tested fractions and selected individual alkaloid standards. The observed background fluorescence was accounted for during data processing to minimize the contribution of sample autofluorescence to the measured AChE-inhibitory activity.

### 2.3. Fingerprinting of Berberis julianae Fruit Extract by HPLC-ESI-QTOF-MS/MS Methodology

The next step included the selection of an extract that could be further evaluated for its AChE inhibition using the prepared assay. As an example, a fruit extract from *Berberis julianae* was taken into consideration due to its rich portfolio of secondary metabolites of different types.

The applied analytical conditions provided high-quality mass spectra. Based on high-resolution mass measurements, retention times, and MS/MS fragmentation patterns, 25 compounds were tentatively identified in the *Berberis julianae* fruit extract. The results are presented in Table 1, while the base peak chromatograms (BPCs) acquired in ESI(−) and ESI(+) modes are provided in the Supplementary Information (Figure S3). The corresponding MS/MS spectra of the tentatively identified compounds are presented in the Supplementary Information (Figure S4).

*Berberis julianae* fruit extract was found to be a rich source of alkaloids and phenolics that were detected and tentatively assigned in the mass spectrometry analysis. As shown in Table 1, a total of eight compounds belonged to the group of isoquinoline alkaloids, confirming the species’ chemotaxonomic affiliation with the Berberidaceae family, which is known for its high content of this alkaloid class. The presence of major alkaloids in barberry fruits has also been confirmed by Abdykerimova et al. (2020) in their study on various organs of *Berberis iliensis* [15].

Additionally, several phenolic acids, flavonoid glucuronides, and glycosides were detected in the extract, consistent with the findings of Och et al., who reported a diverse phenolic profile across various organs of *Berberis vulgaris* [16].

The presence of fatty acid derivatives in *Berberis julianae* fruits reflects their role in plant lipid metabolism, including membrane structure and cuticular wax formation during fruit maturation, which is in line with findings reported by Sun et al., who also identified fatty acids in *Berberis heteropoda* fruits [17]. Despite taxonomic differences among *Berberis* species, similar compounds were identified, indicating that certain phytochemicals are conserved across the genus.

**Table 1:** The list of tentatively assigned components in the studied extract from the fruits of *Berberis julianae* (Rt—retention time, ion mode—ionization, DBE—double bonds and rings equivalent, Δ (ppm)—the difference in the measured *m*/*z*).

№	Molecular Formula	Compound Name	Abbreviation	Ion Mode	Rt (min)	*m*/*z* Observed	*m*/*z* Calculated	Δ (ppm)	DBE	MS/MS Fragments	MS References
1	C_20_H_26_NO_3_^+^	Armepavine or isomer	ARP	+	13.83	328.1915	328.1907	−2.38	9	299, 283,267, 239, 192	
2	C_22_H_21_N_2_O	Unknown 1	UNK1	+	14.08	330.1721	330.1727	1.72	13.5	314, 299, 269	
3	C_21_H_20_N_3_	Unknown 2	UNK2	+	14.41	314.1655	314.1652	−1.04	14	283, 269, 237,	
4	C_23_H_21_N_2_O	Unknown 3	UNK3	+	14.83	342.1725	342.1727	0.48	14.5	297, 282, 265,	
5	C_20_H_24_NO_4_^+^	Magnoflorine	MGF	+	15.49	342.1703	342.1703	−0.92	10	297, 265, 237, 219	[18]
6	C_37_H_40_N_2_O_6_	Berbamine/oxyacanthine or isomer	BBM	+	16.33	609.2983	609.2959	−3.92	19	580, 568, 303	
7	C_21_H_38_N_3_O_8_	Unknown 4	UNK4	+	16.74	461.2737	461.2732	−1.16	4.5	445, 303	
8	C_19_H_18_NO_4_^+^	Demethyleneberberine	DMB	+	17.08	324.1236	324.1230	−1.75	12	309, 294, 280, 266	[19]
9	C_38_H_42_N_2_O_6_	Tetrandrine or isomer	TET	+	17.41	623.3114	623.3116	0.26	19	593, 581, 562, 549	
10	C_20_H_21_NO_4_	Canadine (Tetrahydroberberine)	CND	+	17.74	340.1549	340.1543	−1.67	11	149, 176, 324	[20]
11	C_20_H_20_NO_4_^+^	Jatrorrhizine	JAT	+	18.66	338.1398	338.1387	−3.31	12	323, 308, 294, 280	[20]
12	C_20_H_18_NO_4_^+^	Berberine	BER	+	19.74	336.1232	336.1230	−0.49	13	321, 306, 292, 278	[21]
13	C_7_H_12_O_6_	Quinic acid	QA	-	2.18	191.0563	191.0561	−0.98	2	83	[15]
14	C_16_H_18_O_9_	Chlorogenic acid 1	CGA-1	-	12.35	353.0886	353.0878	−2.24	8	191	[22]
15	C_28_H_30_O_18_	Hesperetin diglucuronide or isomer	HES-DG	-	12.68	653.1374	653.1359	−2.24	14	299, 255, 211, 183	
16	C_16_H_18_O_9_	Chlorogenic acid 2	CGA-2	-	13.43	353.0884	353.0878	−1.68	8	191	[22]
17	C_17_H_20_O_11_	Sinapinic acid-*O*-glucuronide or isomer	SA-GluA	-	13.52	399.0946	399.0933	−3.29	8	367, 251, 179,161	
18	C_20_H_30_O_12_	Verbasoside or isomer	VBS	-	14.35	461.1454	461.1464	2.27	6	416, 191, 149, 131	[23]
19	C_24_H_15_O_4_	Unknown 5	NI-5	-	15.60	367.0977	367.0976	−0.32	17	191, 179, 161, 135	
20	C_27_H_30_O_16_	Rutin (quercetin-3-*O*-rutinoside)	RUT	-	17.35	609.1485	609.1461	−3.92	13	302, 301, 300	[24]
21	C_25_H_24_O_12_	Dicaffeoylquinic acid	DicQA	-	17.85	515.1215	515.1195	−3.88	14	353, 191, 179	[22]
22	C_18_H_34_O_5_	Pinellic acid or isomer	PNL	-	21.27	329.2341	329.2333	−2.28	2	201	
23	C_16_H_32_O_4_	10,16-Dihydroxyhexadecanoic acid	10,16-DHPA	-	21.86	287.2227	287.2228	0.29	1	269, 241, 185, 141	
24	C_15_H_10_O_5_	Galangin	GLN	-	22.61	269.0459	269.0455	−1.31	11	225	[17]
25	C_18_H_30_O_2_	γ-Linolenic acid	GLA	-	24.28	277.2184	277.2173	−3.94	4	233, 205	[25]

### 2.4. CPC Isolation Procedure

In this experiment, pH-zone refining countercurrent chromatography (pH-ZRP CPC) was used to fractionate the extract of interest. In this technique the compounds in the mixture partition between the two phases based not only on their partition coefficients but also on their ionization states, which are influenced by the pH gradient. This method allows for a clearer separation than traditional CPC [26]. pH-ZRP CPC is particularly effective for alkaloids, which the *Berberis* genus is rich in, and can be efficiently separated from other substances in the mixture based on their ionization and solubility properties. Thanks to the application of the technique, twelve pooled fractions were obtained (see Figure S5 with the total chromatogram) and subjected to bioactivity testing.

### 2.5. Determination of Inhibitory Properties of Bereberis julianae CPC Fractions in FAIA

**Figure 3:**
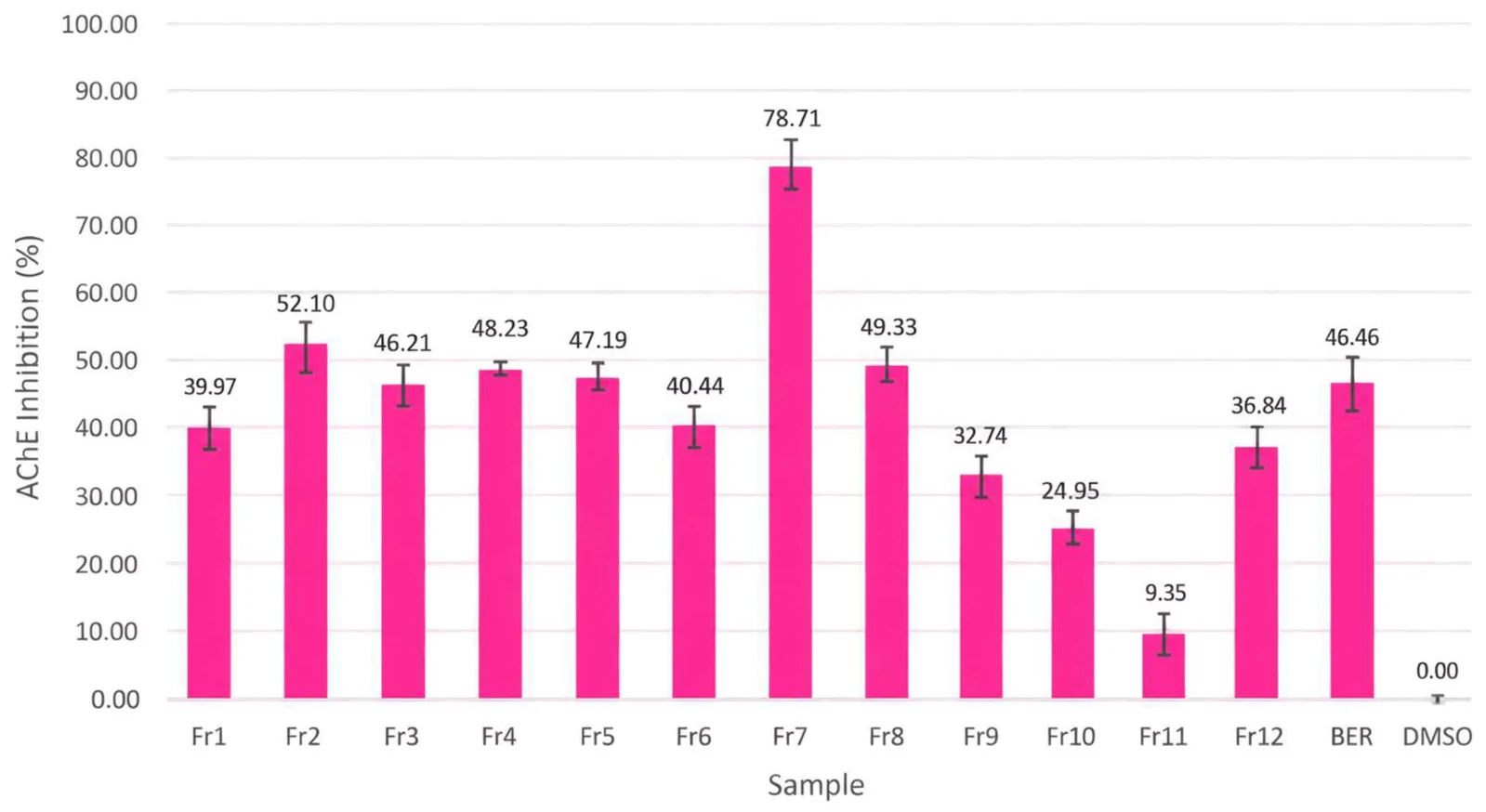
AChE-inhibitory activity of CPC fractions obtained from *B. julianae* fruit extract tested at a working concentration of 5 mg/mL. Berberine (BER, 1 mg/mL) was used as the reference standard. Error bars represent standard deviations (SDs) calculated from three replicate measurements of the samples including background correction (*n* = 3).

An acetylcholinesterase inhibition assay was applied to determine the inhibitory properties of the CPC fractions isolated from the *Berberis julianae* fruit extract. The fractions were tested at a working concentration 5 mg/mL, whereas BER was tested at 1 mg/mL. Fraction 7 showed the highest AChE-inhibitory activity (Figure 3). In the next test, two concentrations (5 and 2 mg/mL) of fraction 7 were compared (see Figure S2 in Supplementary Materials). The measured inhibition percentages were 78.71 ± 1.72% and 44.83 ± 3.21%, respectively. The observed differences in inhibitory activity among the CPC fractions indicate that the fractionation procedure efficiently separated constituents according to their biological activity. Most fractions exhibited moderate or low inhibition, whereas fraction 7 showed distinctly superior activity, suggesting enrichment in the principal AChE-inhibitory constituents of the extract.

The reduction in inhibitory activity observed at lower concentrations of fraction 7 further confirmed the concentration-dependent nature of the biological response and supported the reproducibility of the FAIA measurements. Also, unlike some conventional colorimetric assays, the fluorescence-based readout enabled direct evaluation of alkaloid-rich fractions without visible interference from sample coloration, which is particularly advantageous when screening complex botanical matrices.

### 2.6. The HPLC-MS Fingerprinting of the Fractions from CPC

The enrichment of fraction 7 in the protoberberine alkaloids strongly correlated with its superior AChE-inhibitory activity observed in FAIA, supporting the suitability of the developed workflow for bioactivity-guided isolation of active constituents.

**Figure 4:**
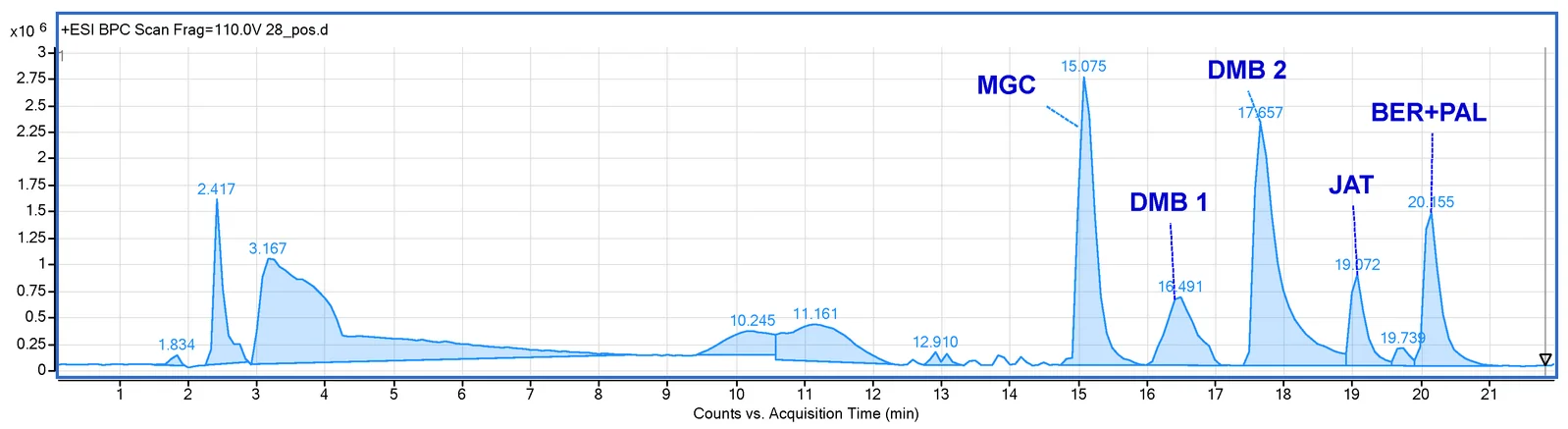
LC-MS base peak chromatogram (BPC) of the analyzed fraction in positive ionization mode (ESI^+^, 110 V) with identified alkaloids.

Its composition can explain the fraction’s inhibitory properties. In the HPLC-MS analysis, four isoquinoline alkaloids were assigned as the major constituents of this fraction: MGC—magnocurarine (RT: 15.08 min), DMB 1, DMB 2—demethyleneberberine isomers (RT: 16.49 and 17.56 min), JAT—jatrorrhizine (RT: 19.37 min), BER—berberine and PAL—palmatine (co-eluting at RT: 20.15 min) (Figure 4). The predominance of protoberberine alkaloids in the most active fraction is consistent with previous reports describing this structural class as one of the most potent groups of naturally occurring AChE inhibitors. The agreement between the chemical profile and the biological activity further validates the usefulness of the proposed FAIA-CPC workflow.

Figure S7 in the Supplementary Material presents the MS/MS spectra of the identified alkaloids (MGC, DMB 1, DMB 2, JAT, BER, and PAL) detected in the analyzed CPC fraction, along with the proposed MS/MS fragmentation pathways. The proposed pathways illustrate possible cleavage patterns of the compounds based on characteristic fragmentation reported in the literature [27,28].

**Figure 5:**
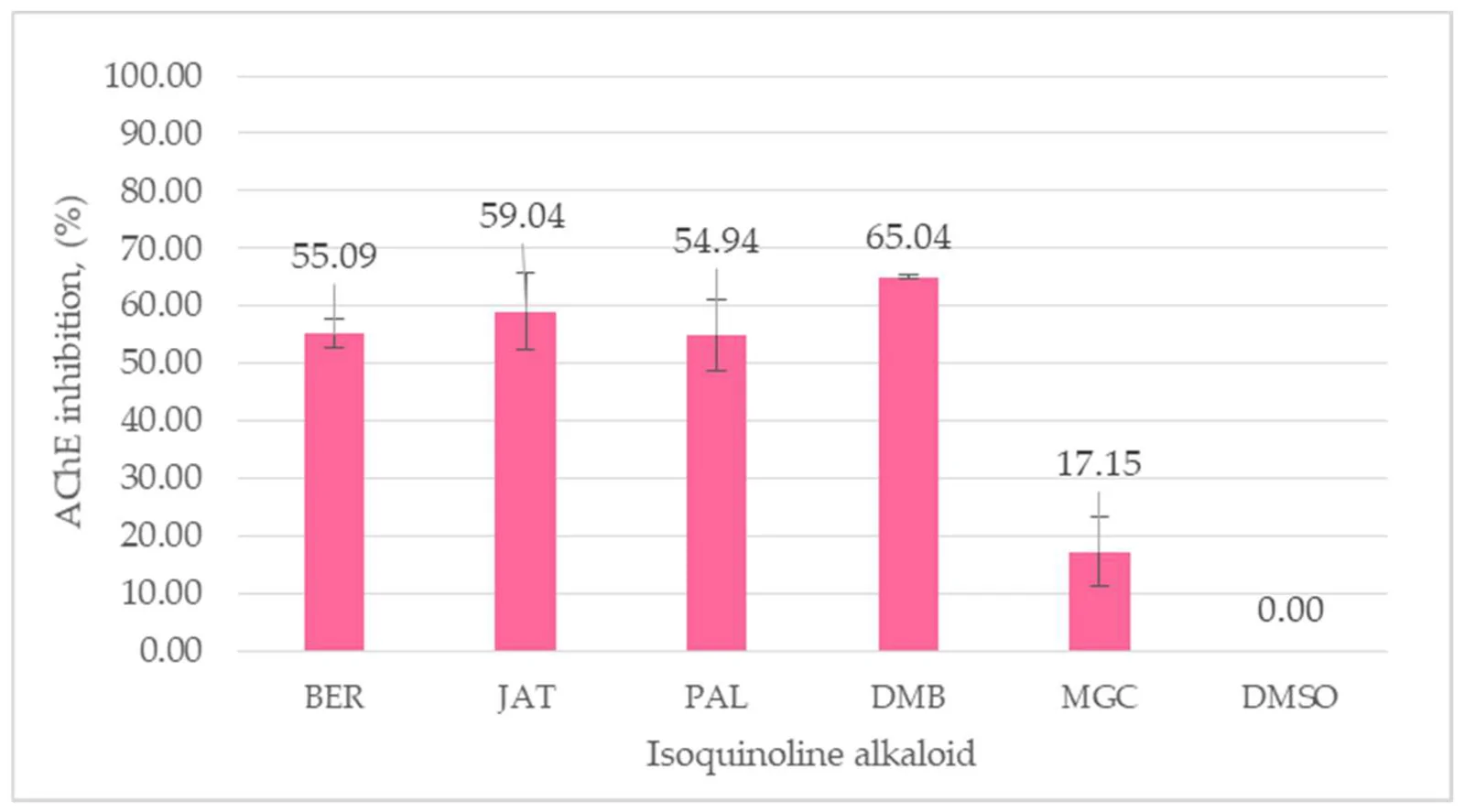
AChE-inhibitory activity of isoquinoline alkaloid standards previously identified in the most active CPC fraction of *B. julianae* fruits. Error bars represent standard deviations (SDs) calculated from three replicate measurements (*n* = 3).

The isoquinoline alkaloids identified by HPLC-ESI-QTOF-MS/MS in the most active fraction of *Berberis julianae* fruits were evaluated for their inhibitory properties using the FAIA (Figure 5). The assay was performed with analytical standards of the same compounds, that is palmatine, magnocurarine, jatrorrhizine, demethylenberberine, and berberine—each at a concentration of 0.0833 mg/mL in a well.

**Figure 6:**
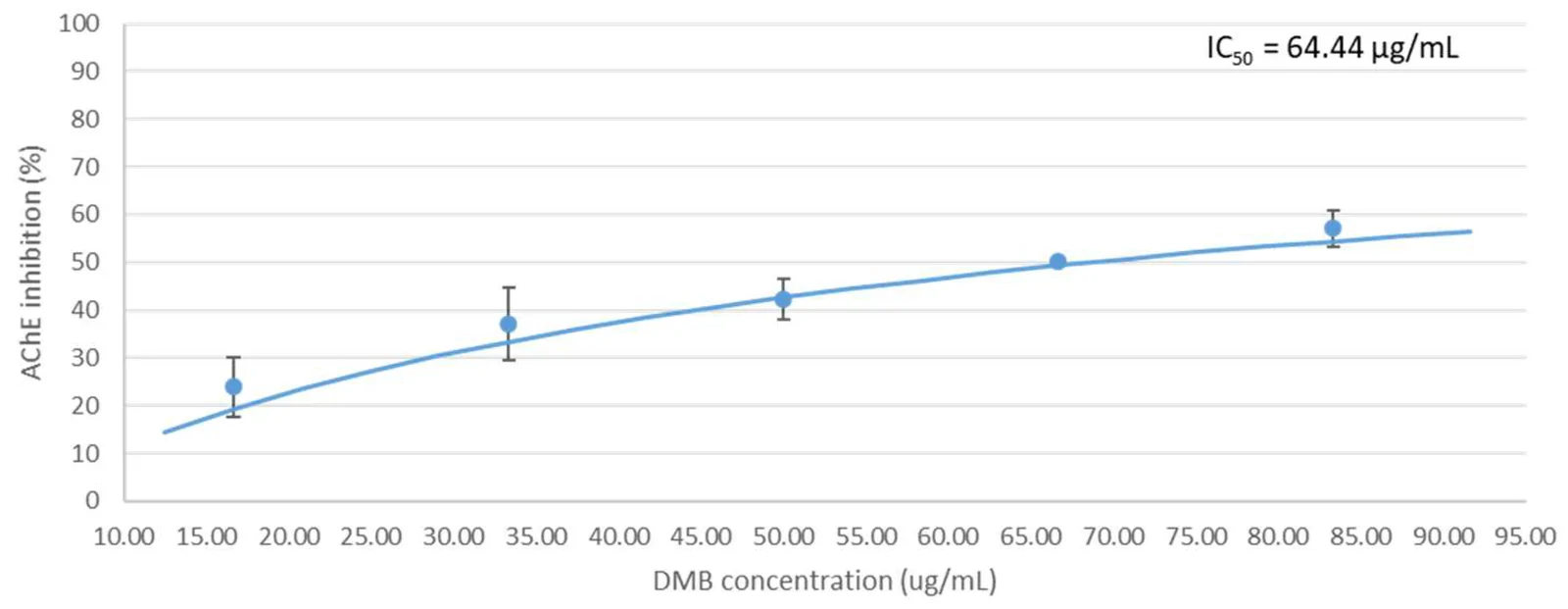
Concentration-dependent inhibitory effect of DMB on AChE activity and determination of the IC_50_ value. Data are presented as mean ± SD (*n* = 3). The fitted concentration–response curve was used to determine an IC_50_ value of 64.44 µg/mL (approximately 199 µM).

Among the tested isoquinoline alkaloids, demethylenberberine (DMB) exhibited the strongest AChE-inhibitory activity, 65.04 ± 0.45% at the tested concentration, followed by jatrorrhizine (JAT), 59.04 ± 6.71%, berberine (BER), 55.09 ± 2.54%, and palmatine (PAL), 54.94 ± 6.17%. Magnocurarine (MGN) showed a weak inhibitory effect of 17.15 ± 6.0%. These results suggest that the AChE-inhibitory activity of the most active CPC fraction (Fraction 7) may be affected by the presence of protoberberine alkaloids. However, the observed activity may also result, at least in part, from synergistic interactions between the compounds present in this fraction, which cannot be excluded. The IC_50_ value was determined for the most active component, DMB, and was found to be 64.44 µg/mL (approximately 199 µM), indicating a concentration-dependent inhibitory effect on AChE activity (Figure 6).

The developed FAIA enabled rapid identification of the biologically most active CPC fraction, which was subsequently confirmed by LC-MS analysis to be enriched in protoberberine alkaloids. The close agreement between fluorescence-based screening and metabolomic characterization demonstrates that the assay was successful for bioactivity-guided fractionation of complex botanical extracts. This workflow simplifies the prioritization of fractions for detailed phytochemical analysis and may facilitate the discovery of novel natural acetylcholinesterase inhibitors from medicinal plants.

### 2.7. Bioassays via TLC and Microplates

The bio tests could be conducted with the help of thin-layer chromatography (TLC) or microplates.

TLC AChE inhibition assays consist of applying potential inhibitors to the plate, eluting them with a solvent system, and then incubating them with a solution containing AChE and the substrate. The plate is visualized under UV light or in an iodine chamber. The intensity of the color at the spots where inhibitors were applied is compared with that of the control spots (without inhibitors). The presence of inhibitors results in reduced or no color development at the spot, indicating enzyme inhibition. This method is less sensitive than other methods, such as spectrophotometric or fluorometric assays, but requires minimal equipment and reagents. Of course, this assay was modified to enhance its sensitivity, specificity, and overall performance.

Microplate AChE inhibition assays are also widely used to screen and quantify compounds, and in this bio test, a 96-well plate is typically used. It requires more equipment than a TLC assay: a spectrophotometer or microplate reader and pipettes are mandatory for performing the test. The procedure starts with preparing solutions: enzyme solution, substrate solution, Ellman’s reagent, and solutions of the tested compounds. The enzyme and potential inhibitors are applied to the test plate and incubated for a specific time. Afterward, add substrate solution and Ellman’s reagent to each well. The plate is incubated again for some time. Measure the absorbance at 412 nm using a microplate reader. Microplate assays allow the simultaneous screening of many compounds and provide accurate measurements at low levels of enzyme activity.

### 2.8. CPC Fraction Cytotoxicity in SH-SY5Y Cells

**Figure 7:**
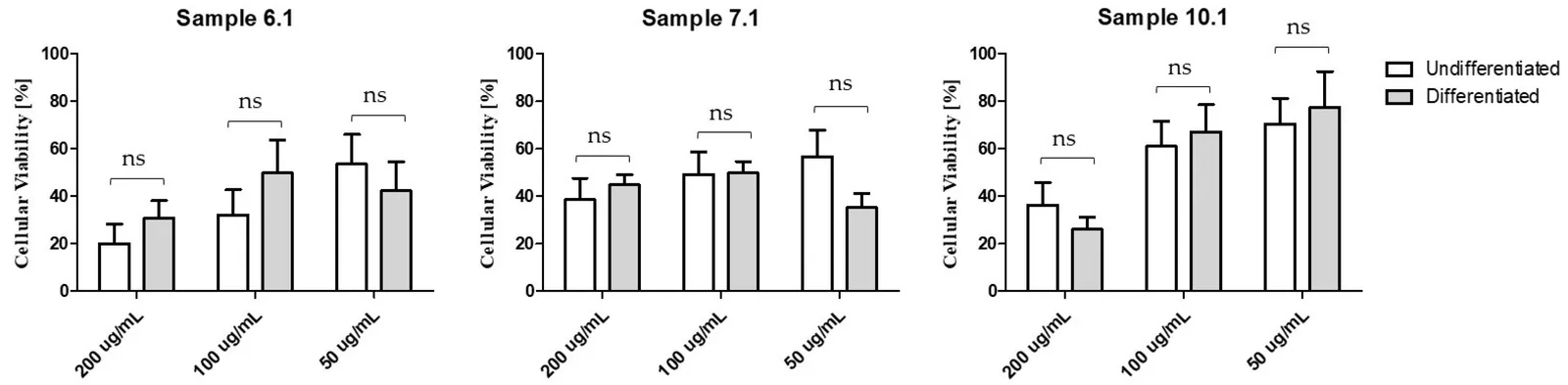
Cytotoxicity of selected CPC fractions from *Berberis julianae* fruits in differentiated and undifferentiated SH-SY5Y cells after 24 h exposure. Cell viability is expressed as the percentage relative to the untreated control (mean ± SD). Data are presented as mean ± SD; *n* = 3. Here, *n* = 3 denotes three independent biological experiments (independently seeded, differentiated, and treated cultures), with technical replicate wells averaged within each experiment before statistical analysis; for fraction 7 in differentiated cells, *n* = 1 (see Section 2.7 for details). Two-way ANOVA performed statistical comparisons; ns—not significant.

To investigate the relationship between alkaloid composition and cytotoxic activity, three CPC fractions with distinct phytochemical profiles were evaluated for cytotoxicity in differentiated and undifferentiated SH-SY5Y cells using the Alamar Blue reduction assay following 24 h of exposure (Figure 7). Fraction 7 represented the most active AChE-inhibitory fraction, whereas fraction 6 exhibited a closely related composition, differing mainly by the presence of magnoflorine and the absence of palmatine. Fraction 10, in turn, was characterized by a distinct bisbenzylisoquinoline alkaloid profile, including berbamine/oxyacanthine isomers, tetrandrine isomers, amperavine, and one unidentified compound (see Figure S8 in Supplementary Material).

It exhibited concentration-dependent cytotoxicity toward SH-SY5Y cells, with cell viability generally increasing as the concentration decreased. Among the tested fractions, fraction 10 exhibited the highest cell viability and, therefore, the lowest cytotoxicity in both differentiated and undifferentiated SH-SY5Y cells, at 50 and 100 μg/mL. Notably, at the highest tested concentration (200 μg/mL), differentiated SH-SY5Y cells treated with fraction 7 displayed significantly higher viability than those treated with fraction 10 (44.7 ± 4.4% vs. 26.0 ± 5.2%; *p* = 0.009), whereas no significant difference between these two fractions was observed in undifferentiated cells at this concentration (38.5 ± 9.1% vs. 36.2 ± 9.5%; *p* = 0.78). This finding suggests that the relative cytotoxic ranking of fractions 7 and 10 is not fully consistent across the tested concentration range and may reflect concentration- and differentiation-status-dependent, non-additive interactions among the phytochemical constituents of these complex fractions.

It should be noted that the fraction 7 dataset for differentiated cells at 200 μg/mL is derived from a single independent experiment (technical triplicate); owing to the limited amount of fraction 7 obtained, the other two of the three planned independent experiments could not be performed for this specific sample. This comparison should therefore be regarded as exploratory rather than a fully powered biological-replicate comparison, and independent confirmation is planned. For this reason, fraction 7 in differentiated cells (*n* = 1) was excluded from the two-way ANOVA reported in Section 2.8 and Section 4.11. Experiments with protoberberine alkaloid standards and differentiated SH-SY5Y cells did not consistently exhibit greater cytotoxicity than undifferentiated cells after.

### 2.9. Protoberberine Alkaloids Cytotoxicity in SH-SY5Y Cells

**Figure 8:**
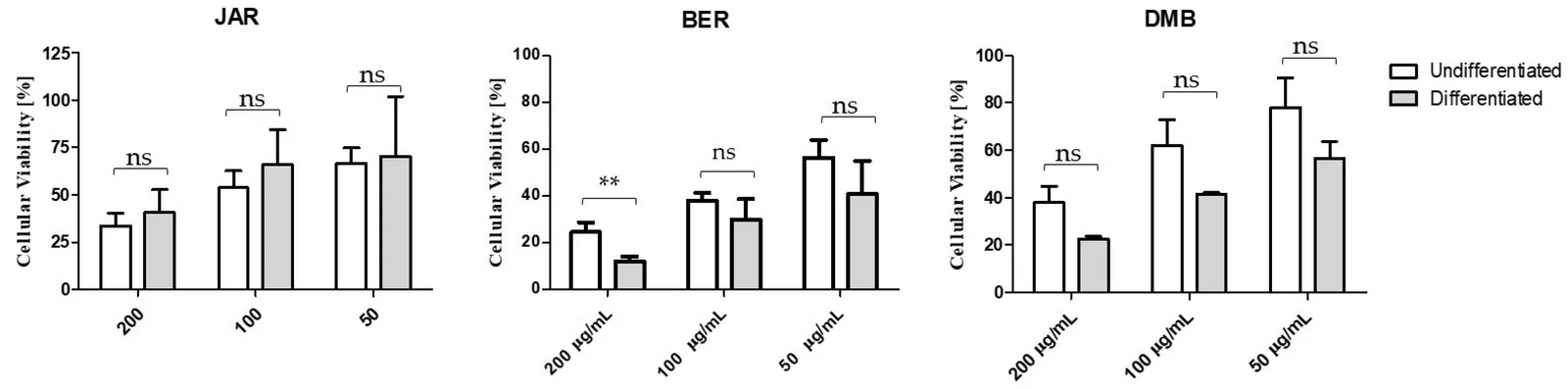
Concentration-dependent cytotoxicity of protoberberine alkaloids jatrorrhizine (JAR), berberine (BER) and demethyleneberberine (DMB) in undifferentiated and differentiated SH-SY5Y cells. Results are expressed as the percentage of viable cells relative to the untreated control (mean ± SD, *n* = 3). Here, *n* = 3 denotes three independent biological experiments, with technical replicate wells averaged per experiment. For jatrorrhizine (JAR) in differentiated cells, *n* = 2: one of three experiments was excluded owing to a documented culture-specific technical failure (see Section 2.8). Two-way ANOVA performed statistical comparisons; ns—not significant; ** *p* < 0.01.

Three protoberberine alkaloids, jatrorrhizine, berberine, and demethyleneberberine, were tested for cytotoxicity in SH-SY5Y cells using the Alamar Blue reduction assay after 24 h of exposure at concentrations of 50–200 μg/mL (Figure 8).

Among the tested alkaloids, berberine exhibited the strongest cytotoxic effect, consistently reducing cell viability to a greater extent than jatrorrhizine and demethyleneberberine at corresponding concentrations. In contrast, demethyleneberberine showed the lowest cytotoxicity, whereas jatrorrhizine displayed an intermediate effect. Two-way ANOVA (concentration × differentiation state) confirmed a significant effect of concentration for berberine, demethyleneberberine and jatrorrhizine (berberine and demethyleneberberine: *p* < 0.0001; jatrorrhizine: *p* = 0.039), a significant effect of differentiation state for berberine (*p* = 0.004) and demethyleneberberine (*p* = 0.003) but not jatrorrhizine (*p* = 0.39), and no significant concentration × differentiation-state interaction for any of the three alkaloids (*p* > 0.08). For jatrorrhizine, one of three independent experiments in differentiated cells was excluded from this analysis (*n* = 2) owing to a documented culture-specific technical failure unrelated to treatment concentration. All protoberberine alkaloids exhibited concentration-dependent cytotoxicity, with cell viability decreasing as the concentration increased. In undifferentiated cells, among the tested alkaloids, BER exhibited the strongest cytotoxic effect, consistently reducing cell viability to a greater extent than JAR and DMB at the corresponding concentrations. In contrast, DMB showed the lowest cytotoxicity. Differentiated SH-SY5Y cells were more susceptible to the cytotoxic effects than undifferentiated cells, which may be explained by differentiation-induced changes in cellular metabolism, resulting in increased energetic stress, oxidative vulnerability, and decreased mitochondrial membrane potential [29]. Previous studies have demonstrated that berberine induces mitochondrial dysfunction in primary neurons, characterized by mitochondrial swelling, increased oxidative stress, decreased mitochondrial membrane potential, and ATP depletion [30]. These findings suggest that mitochondrial dysfunction may be one of the mechanisms underlying the increased susceptibility of differentiated SH-SY5Y cells to protoberberine alkaloids observed in the present study.

## 3. Discussion

### 3.1. Berberine Inhibitory Effect Comparison in Fluorescence Acetylcholinesterase Inhibition Assay (FAIA)

The present study demonstrates that FAIA can be successfully applied to the rapid screening of complex botanical samples. Compared with the classical Ellman assay, the proposed method requires fewer reagents, eliminates interference associated with chromogenic detection, and can be readily implemented in a standard fluorescence microplate format. Validation with berberine demonstrated that the assay reliably detected AChE inhibition and exhibited sensitivity comparable to established colorimetric methods. These characteristics make FAIA particularly suitable for bioactivity-guided fractionation workflows, where numerous fractions obtained during phytochemical isolation must be screened rapidly before detailed chemical characterization.

Plant-derived alkaloids play an important role in medicine, as many are widely used drugs for treating various ailments. Among the compounds in this metabolite group, berberine, galantamine, and huperzine A have been shown to exhibit acetylcholinesterase (AChE) inhibitory activity [31,32,33,34].

**Figure 9:**
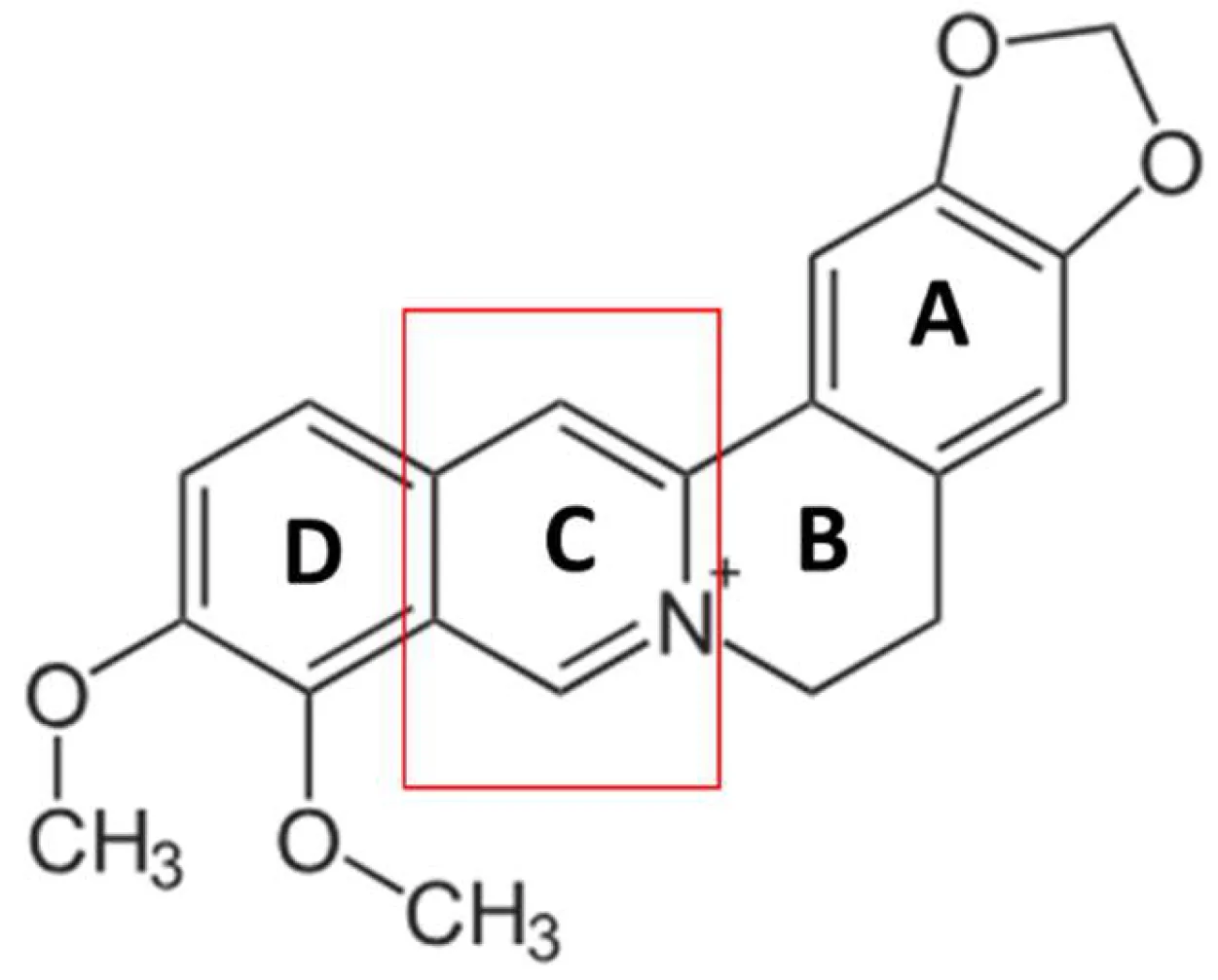
Chemical structure of berberine. The red box highlights the structural moiety crucial for AChE inhibition.

Berberine (BER) is an isoquinoline alkaloid of the protoberberine group, detected most often in the barberry genus. High concentrations of this alkaloid are in the roots, rhizomes, stems, and bark of *Berberis*; less of it is found in the leaves and fruits. As mentioned above, BER is a natural inhibitor of AChE [35]. Although BER has been reported to cross the blood–brain barrier and exert neuroprotective effects, its brain exposure is limited by its poor bioavailability and pharmacokinetic properties [36,37,38]. The ring C in the conjugated structure of BER is responsible for AChE inhibition; the quaternary ammonium group can interact with the anionic site of acetylcholinesterase (Figure 9). Metabolic changes, such as hydrogenation in ring C, lead to a decrease in inhibitory properties of the compound [39].

This alkaloid was selected as a reference to optimize the fluorimetric assay parameters described in this publication.

Similar results from AChE inhibition were reported in an article comparing combined drug therapy for the treatment of Alzheimer’s disease and are comparable to those observed in our study [40]. The authors determined the level of AChE inhibition of four drugs. AChE from *Torpedo californica* was incubated with donepezil, tacrine, berberine, and galantamine in an Ellman assay performed on microtiter plates. The results of the experiment showed that the activity of the substances decreased in the following order: donepezil > tacrine > berberine > galantamine. The IC50 values for BER and GAL were 3234 nM and 4173 nM, respectively, in the assay. Based on these data, BER was selected as the standard substance for the FAIA. In the present work, this workflow enabled the efficient identification of the most active CPC fraction of *Berberis julianae*, which was subsequently characterized by HPLC-MS and shown to be enriched in protoberberine alkaloids. Thus, FAIA represents not only an analytical assay but also a practical screening platform facilitating natural-product-based discovery of new acetylcholinesterase inhibitors.

### 3.2. Potential Role of Identified Isoquinoline Alkaloids in AChE Inhibition

The diverse group of natural compounds known as isoquinoline alkaloids shows potential for combating Alzheimer’s disease (AD) by targeting multiple pathways implicated in the disease. According to various sources in the literature, they have been investigated through in vitro, in vivo, and in silico studies. In the study by de Almeida et al., erythraline and erysodine delayed the onset of Aβ_1_–_42_-induced toxicity in vivo using the transgenic *C. elegans* CL2006 model, suggesting their potential as neuroprotective agents for Alzheimer’s disease [41].

**Figure 10:**
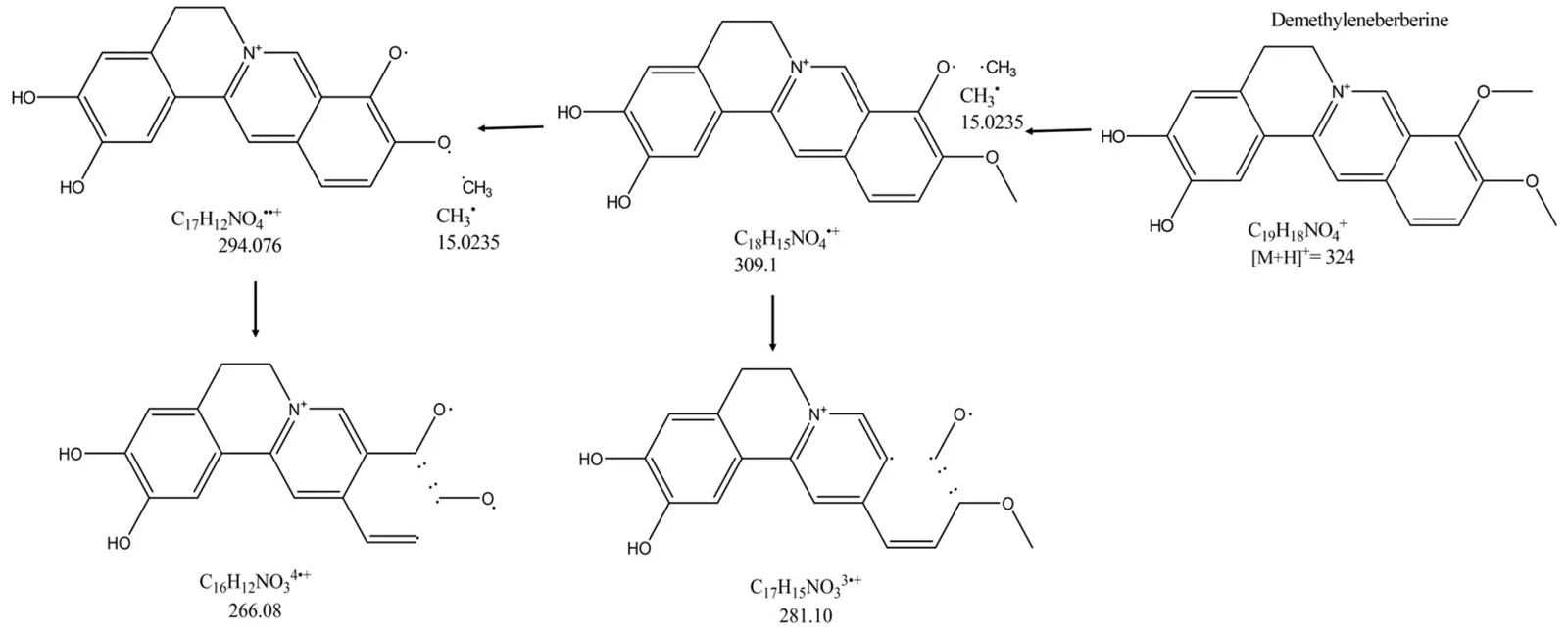
Proposed MS/MS fragmentation pathway of demethyleneberberine.

The alkaloids identified in the potentially active CPC fraction of *Berberis julianae* fruits, including demethyleneberberine, jatrorrhizine, and palmatine, belong to the protoberberine class of alkaloids and are structurally related to berberine. Due to their common protoberberine skeleton, these compounds may exhibit similar MS/MS fragmentation behavior. The proposed fragmentation pathways were therefore interpreted based on the characteristic fragmentation behavior of BER, as reported in the literature [42], including the losses of CH_3_•, CH_4_, and CO. The proposed MS/MS fragmentation pathway of DMB is presented in Figure 10, while the proposed fragmentation pathways of the other identified protoberberine alkaloids are provided in the Supplementary Materials (Figure S10).

Demethyleneberberine (DMB) is an active BER metabolite that exhibits pharmacological effects similar to those of BER but has improved blood–brain barrier-crossing capacity [43].

Jatrorrhizine and palmatine have some structural similarities: jatrorrhizine differs only in the hydroxide group in one of the radicals of ring A—this change causes a reduction in inhibitory activity against AChE [44].

**Figure 11:**
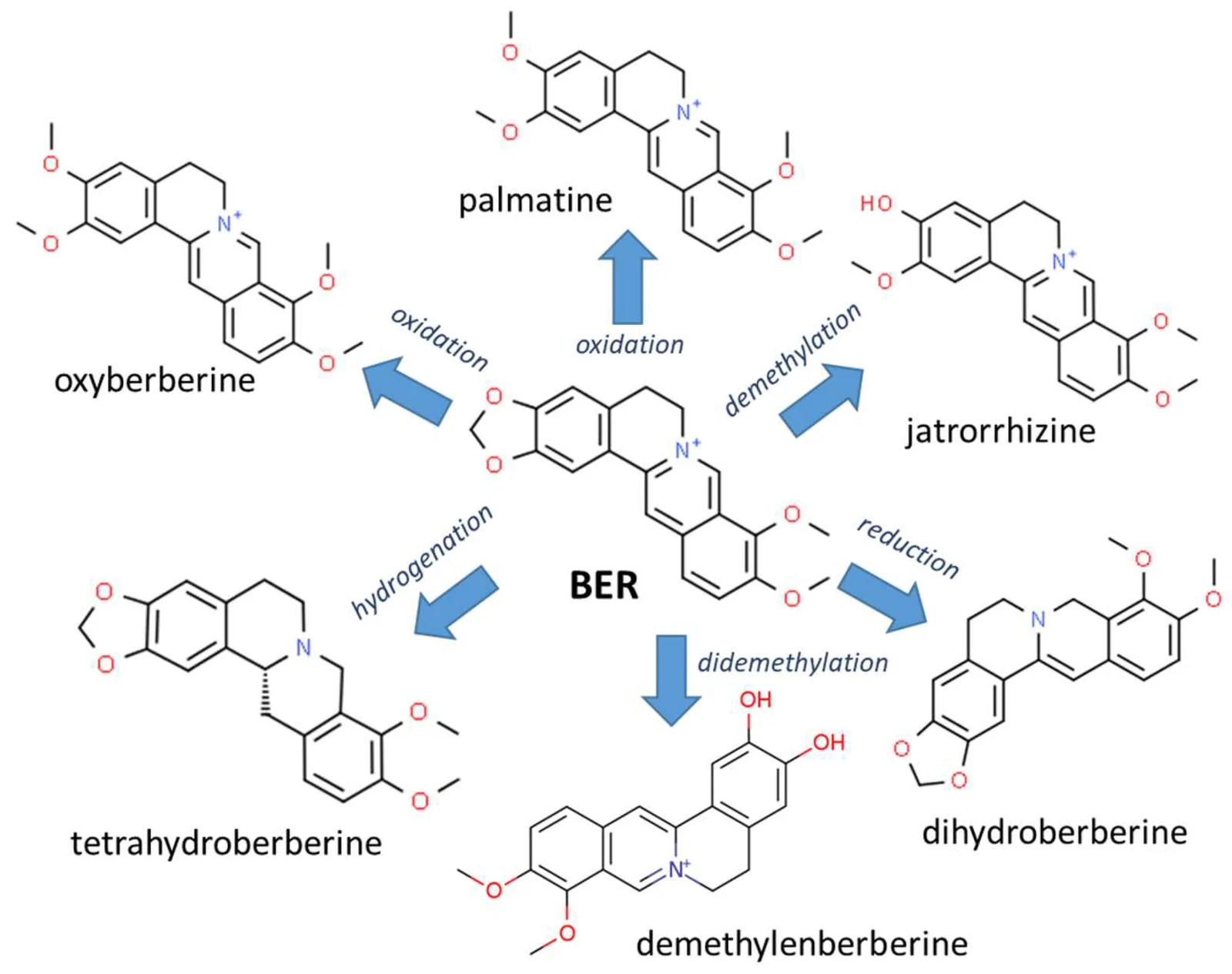
Protoberberine alkaloids and a possible plant pathway of their formation.

The identified protoberberine alkaloids are structurally closely related and may be interconnected through biosynthetic transformations occurring in plants (Figure 11). Berberine represents a central intermediate from which other protoberberine alkaloids can be formed through reactions such as hydroxylation, O-demethylation, reduction, oxidation, or hydrogenation. In the analyzed *B. julianae* fruit extract, the simultaneous occurrence of berberine, palmatine, jatrorrhizine, and demethyleneberberine is consistent with their common biosynthetic origin. This structural relationship may also explain the similarities observed in their MS/MS fragmentation patterns and biological activity.

**Figure 12:**
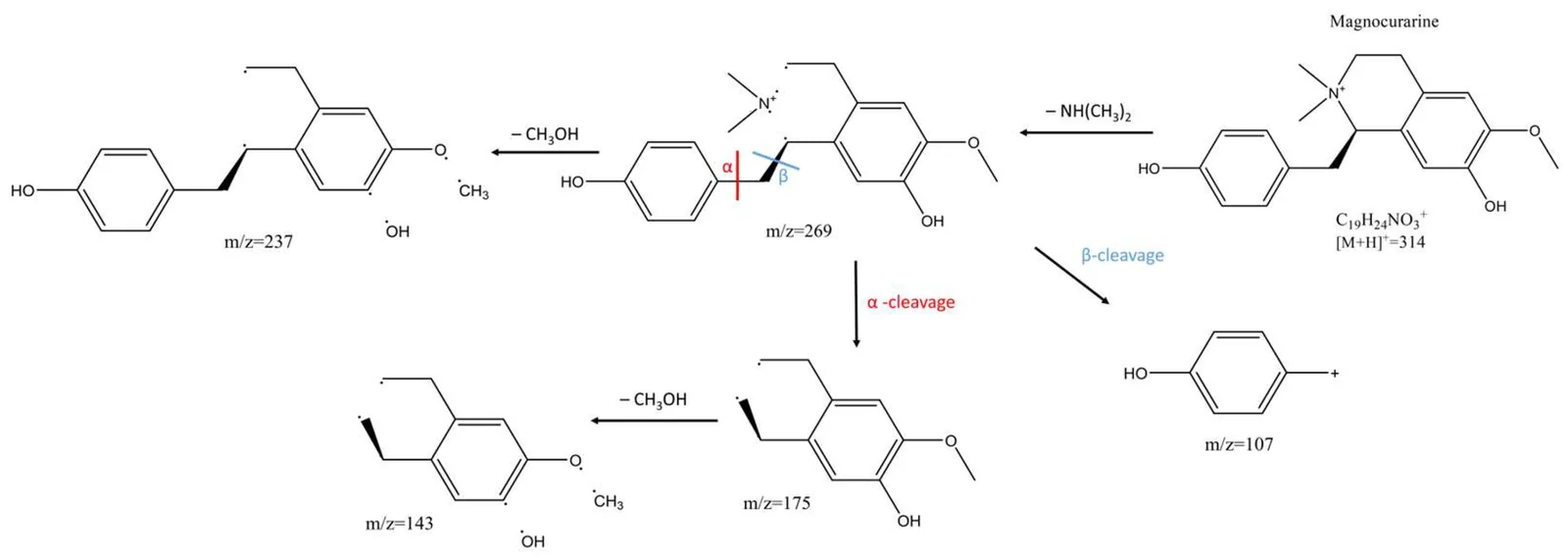
Proposed MS/MS fragmentation pathway of magnocurarine.

Magnocurarine belongs to the benzylisoquinoline-benzyl-tetrahydroisoquinoline type of alkaloids. This type of structure in MS fragmentation can lose –NH(CH_3_)_2_, –NH_2_CH_3_, or –NH_3_, as in aporphine-type alkaloids. The latter occurs via cleavage of the alkaloid skeleton, resulting in mass fragments below *m*/*z* 200. The hydroxyl group at the 4′-position facilitated β-cleavage, yielding a stable aromatic ion at *m*/*z* 107 corresponding to a para-hydroxybenzyl cation. Furthermore, α-cleavage generated a characteristic fragment at *m*/*z* 175. Both –OH and –OCH_3_ groups are electron-donating groups located on ring A of the alkaloid and contribute to the resonance stabilization of the resulting ion, favoring the cleavage pathway [28] (as presented in Figure 12).

Although no studies have directly evaluated magnocurarine as an AChE inhibitor, a related tetrahydrobenzylisoquinoline derivative, namely (S)-2-(1,3-propanediol-2-yl)-isococlaurine, has demonstrated moderate inhibitory activity against AChE, with an IC_50_ value of 8.2 ± 1.8 μM, with the modified Ellman’s method. In addition, computer simulations (docking and molecular dynamics) showed that this compound binds the enzyme well and stably [45].

The benzylisoquinoline alkaloids described above demonstrated the ability to inhibit acetylcholinesterase in different experimental models and with varying potency. In the studied fraction, they act together, probably enhancing or stabilizing each other’s effects. This property underscores their potential therapeutic applications in treating diseases like Alzheimer’s.

### 3.3. Assessment of the AChE-Inhibitory Activity of the Identified Alkaloids

Various studies have reported different levels of AChE inhibition for protoberberine alkaloids, which may be attributed to differences in experimental conditions as well as structural variations within the protoberberine skeleton. The evaluation of protoberberine alkaloids for their AChE-inhibitory activity in the study by Hai-Tao Xiao et al., performed using the microplate assay by Ellman, revealed the following IC_50_ values: berberine—0.47 ± 0.01 µM, palmatine—0.74 ± 0.06 µM, and jatrorrhizine—2.08 ± 0.09 µM. Berberine exhibited higher AChE-inhibitory activity than palmatine, which may be attributed to the presence of a methylenedioxy group at C-2 and C-3. In contrast, jatrorrhizine demonstrated lower activity than palmatine, possibly due to the presence of a hydroxyl group [46]. In another study, Pei Li reported the following order of AChE-inhibitory activity, as determined by the Ellman assay in 96-well microplates: palmatine > jatrorrhizine > berberine. The authors attributed these differences to variations in the substitution pattern at positions C-2, C-3, C-9, and C-10 [47]. To date, no scientific data are available on the AChE-inhibitory potential of demethylenberberine. Notably, in the present study, demethylenberberine demonstrated the highest AChE-inhibitory activity among all tested alkaloids. Molecular docking confirms the influence of substituents on the molecular flexibility and interaction with the enzyme pocket. In addition, the aromaticity of the molecules plays a crucial role, as it enables the formation of stable π–π interactions with the enzyme’s aromatic amino acid residues [48].

The cytotoxic concentration causing a 50% reduction in cell viability (CC_50_) was also estimated for DMB and BER from the concentration-dependent cytotoxicity data shown in Figure 6. For DMB, the estimated CC_50_ values were approximately 150 µg/mL for undifferentiated SH-SY5Y cells and 70 µg/mL for differentiated SH-SY5Y cells. Based on the AChE IC_50_ value of 64.44 µg/mL, the corresponding selectivity indices (SI = CC_50_/IC_50_) were approximately 2.3 and 1.1, respectively. For BER, the AChE IC_50_ was 47.51 µg/mL (approximately 141 µM), while the estimated CC_50_ for undifferentiated SH-SY5Y cells was approximately 67 µg/mL, resulting in an SI of approximately 1.4. In differentiated SH-SY5Y cells, viability was already below 50% at the lowest tested BER concentration (50 µg/mL); therefore, the CC_50_ was estimated as <50 µg/mL, corresponding to an SI < 1.05. Overall, although BER exhibited stronger AChE-inhibitory potency than DMB, as indicated by its lower IC_50_, DMB showed a more favorable selectivity margin, particularly in undifferentiated SH-SY5Y cells. Nevertheless, the relatively low SI values observed for both alkaloids indicate that their AChE-inhibitory concentrations are relatively close to those associated with reduced cell viability, particularly in differentiated cells. Therefore, their AChE-inhibitory activity should be interpreted in conjunction with their concentration-dependent cytotoxicity. The inhibitory concentrations obtained using FAIA were higher than some previously reported values determined using the Ellman assay. Since IC_50_ is an assay-dependent parameter, it may be influenced by several experimental factors, including the source and concentration of AChE, the nature and concentration of the substrate, inhibitor pre-incubation time, reaction time, buffer composition, and the method used for activity detection [49]. Present FAIA employs 4-methylumbelliferyl acetate (4-MUA) as a fluorogenic substrate, whereas the Ellman method is generally based on acetylthiocholine. Since these substrates differ in their chemical structures, they may exhibit different enzyme–substrate interactions, thereby affecting the apparent inhibitory behavior of AChE inhibitors. Consistent with this assumption, Berman and Leonard demonstrated that AChE-inhibitory behavior varied with the substrate used, with acetylthiocholine exhibiting relatively simple mixed inhibition patterns. In contrast, the uncharged substrate 4-MUA exhibited more complex, nonlinear inhibition patterns in the presence of cationic ligands [50]. Thereby, IC_50_ values obtained using FAIA should not be considered directly interchangeable with values generated using the Ellman assay under different experimental conditions. To the best of our knowledge, this is the first application of 4-MUA as a fluorogenic substrate in this type of microplate-based AChE inhibition assay; therefore, substrate-specific effects on the apparent inhibitory potency of AChE inhibitors remain insufficiently characterized. For screening applications, the interpretation of inhibitory activity should therefore also consider the relative response of test compounds within the same assay system and in comparison with a reference inhibitor with well-established biological activity. This approach may be more informative for bioactivity-guided screening than a direct comparison of absolute IC_50_ values obtained using different assay formats. This is particularly relevant for AChE inhibitors, for which considerable variation in reported inhibitory potency and even in the relative ranking of structurally related alkaloids can be observed between studies. In the present study, berberine was used as a reference inhibitor because of its well-documented AChE-inhibitory activity, and the FAIA provided a reproducible concentration-dependent response to this compound. Therefore, although the absolute IC_50_ values obtained using FAIA may differ from those reported using the Ellman assay, the method remains useful for assessing the relative inhibitory potential of samples under standardized assay conditions and for guiding the selection of fractions or compounds for subsequent investigation.

In this study, all three tested protoberberine alkaloids exhibited concentration-dependent cytotoxicity toward SH-SY5Y cells in the studied concentration range (25–200 μg/mL), as evidenced by a progressive decrease in cell viability with increasing concentration. To date, the cytotoxicity of berberine has been investigated in SH-SY5Y cells only to a limited extent. Kim et al. evaluated berberine at a single concentration (75 μg/mL) in combination with arsenic trioxide (As_2_O_3_) and demonstrated that it enhanced As_2_O_3_-induced apoptosis through increased ROS production, caspase-3 activation, and downregulation of anti-apoptotic proteins. However, this study investigated the synergistic effect of berberine with a chemotherapeutic agent rather than its intrinsic cytotoxicity toward SH-SY5Y cells [51]. Berberine at sub-cytotoxic concentrations (1–10 μM) attenuated rotenone-induced neurotoxicity by restoring cell viability, reducing ROS production, recovering mitochondrial membrane potential, and reactivating the PI3K/Akt signaling pathway. However, the intrinsic cytotoxicity of berberine was not investigated, as only neuroprotective concentrations were employed [52]. Among the available studies, only one directly evaluated the intrinsic cytotoxicity of berberine in undifferentiated SH-SY5Y cells using the MTT assay. Berberine exhibited a concentration-dependent effect: at 30 μg/mL it exerted neuroprotective activity against H_2_O_2_-induced oxidative stress, whereas at higher concentrations (150–300 μg/mL) it significantly reduced cell viability, indicating intrinsic cytotoxicity [53]. Thus, none of the identified publications evaluated the cytotoxicity of berberine in RA-differentiated, post-mitotic SH-SY5Y cells or compared the responses of differentiated and undifferentiated cells. Moreover, to the best of our knowledge, the cytotoxicity of jatrorrhizine and demethyleneberberine has not yet been investigated in SH-SY5Y cells.

This study has several limitations. Cytotoxicity was assessed using the resazurin (AlamarBlue) reduction assay, which reports cellular metabolic/reductase activity as a surrogate marker of viability rather than directly measuring cell death. Independent cell death assays (e.g., LDH release, Annexin V/PI staining, or live/dead staining) were not performed and could further clarify the mode of cell death underlying the observed decreases in viability. In addition, cell-free control experiments were not performed to formally exclude direct interference of the tested alkaloids and fractions with the redox chemistry or fluorescence of the resazurin/resorufin system. Future studies should incorporate these complementary assays to strengthen the mechanistic interpretation of the cytotoxicity data. Another limitation of the present study is the use of AChE derived from *Electrophorus electricus* and not human AChE. Although this enzyme is widely used for screening and characterizing AChE inhibitors, species-specific differences in enzyme structure and inhibitor interactions may affect inhibitory potency. Therefore, the activity observed in the present study should be considered primarily as screening evidence, and further studies using human AChE are required to confirm the inhibitory effects and assess their translational relevance.

## 4. Materials and Methods

### 4.1. Reagents

The following reagents were used for CPC: methyl tert-butyl ether, *n*-butanol, and acetonitrile, all purchased from Avantor Performance Materials (Gliwice, Poland). LC-MS-grade water and acetonitrile were purchased from Merck (Darmstadt, Germany). Reagents used for the fluorescence assay included acetylcholinesterase from *Electrophorus electricus* (electric eel), Trizma base, bovine serum albumin (BSA), 4-methylumbelliferyl acetate (4-MUA), and berberine, all purchased from Sigma-Aldrich (St. Louis, MO, USA). Dimethyl sulfoxide (DMSO) and ethanol were of analytical reagent grade. Redistilled water was used throughout the experiments. Isoquinoline alkaloid standards analyzed using FAIA included palmatine chloride (Sigma-Aldrich, St. Louis, MO, USA), magnocurarine (ChemFaces, Wuhan, China), jatrorrhizine (TRC Canada, North York, ON, Canada), and demethylenberberine (Sigma-Aldrich, St. Louis, MO, USA).

### 4.2. Plant Material

Fruits of *Berberis julianae* used in this study were collected in Kyiv, Ukraine, at the O.V. Fomin Botanical Garden of the Taras Shevchenko National University of Kyiv in September 2022. The fruits were authenticated by Myroslav Shevera and by Tetiana Dvirna. The plant material was air-dried and ground into a powder using a laboratory mill before further processing. A voucher specimen is stored in the Department of Pharmacognosy of the Medical University of Lublin by the authors of the manuscript.

### 4.3. Preparation of Plant Extracts

One gram of powdered plant material was placed in a Falcon test tube and filled to 15 mL with methanol. The sample was subjected to ultrasound-assisted extraction at room temperature for 30 min. After extraction, the Falcon tubes were centrifuged for 5 min. The resulting extract was transferred to Eppendorf tubes and evaporated to dryness in a concentrator at 40 °C. The procedure was performed three times on three separate portions of fruits. The dried residue was stored in the freezer until the analysis—but no longer than 2 weeks.

### 4.4. HPLC-ESI-QTOF-MS/MS Profiling of the Extract and Fractions

The obtained extracts and fractions were analyzed by HPLC-ESI-QTOF-MS to identify the metabolites present in *Berberis julianae* fruits and to sort the fractions by composition. The chromatographic analysis was performed using an HPLC-ESI-QTOF instrument (6500 Series, Agilent Technologies, Santa Clara, CA, USA), composed of a 1200 Series HPLC equipped with an autosampler (G1329B), binary pump (G1312C), photodiode array detector (DAD, G1315D), degasser (G1322A), column thermostat, and a 6530B QTOF mass spectrometer with an electrospray ionization (ESI) source. For the separation, a Zorbax Eclipse Plus RP-18 chromatographic column (150 mm × 2.1 mm, 3.5 µm) produced by Agilent Technologies, Santa Clara, CA, USA, was used. The mobile phase was delivered by a two-channel pump: channel A contained water with 0.1% formic acid, whereas channel B contained acetonitrile with 0.1% formic acid. The solvent’s time gradient dependence was 10 min—20%, 15 min—40%, 17–18 min—95%, and 19–25 min—1% acetonitrile with 0.1% formic acid. The flow rate was 0.2 mL/min, the column temperature was maintained at 25 °C, and the injection volume was 20 µL. UV spectra were recorded at 254, 280, 320, and 365 nm within the range of 190–600 nm. Mass spectra were acquired in both positive and negative ionization modes over an *m*/*z* range of 100–1200. The ESI source was operated with a capillary voltage of 3.5 kV, drying gas flow of 12 L/min, drying gas and sheath gas temperatures of 350 and 325 °C, respectively, a fragmentor voltage of 150 V, a skimmer voltage of 65 V, and collision energies of 10 and 20 eV. MS/MS spectra were acquired in data-dependent acquisition mode for the two most intense precursor ions in each scan. After acquisition of one MS/MS spectrum, the corresponding precursor ion was dynamically excluded for 0.2 min to enable fragmentation of less abundant ions. Compound identification was based on MS/MS fragmentation patterns, comparison with authentic reference standards, retention times, data from the literature, and the PubChem database. Data analysis was performed using the Agilent MassHunter Workstation software (version B.10.00).

### 4.5. Fractionation of the Extract

The centrifugal partition chromatograph (CPC) (SCPC-250-L, Armen Instruments, Saint Ave, France), equipped with a 250 mL stainless steel column, a UV detector, a fraction collector, a pump and injection valve with a 10 mL sample loop, was used in the study to fractionate the methanolic extract from *Berberis julianae* fruits. The fractionation of the extract was performed on the initially optimized composition of the biphasic solvent system.

### 4.6. Solvent System Selection

The biphasic solvent system used to fractionate fruit extracts from *B. julianae* was selected based on partition coefficient values (*K*) determined for a set of solvent mixtures reported in the scientific literature using the test tube method (see Table 2 and Supplementary Material, Table S1).

The tested CPC solvent systems were prepared in glass test tubes to a total volume of 5 mL. Subsequently, 20 mg of the crude *B. julianae* fruit extract was added to each system. The samples were mixed thoroughly and filtered through 0.1 µm syringe filters (Merck) into HPLC vials. Aliquots (1 mL) of both the upper and lower phases were collected for LC-MS analysis. Based on the obtained results, solvent system No. 2 (Table 2) was selected for further CPC fractionation.

### 4.7. Preparation of a Biphasic Solvent System

The CPC separation was performed using a biphasic solvent system composed of methyl tert-butyl ether, butanol, acetonitrile, and water (2:2:1:5 *v*/*v*/*v*/*v*). Triethylamine (TEA, 10 mM) was added to the organic phase, while hydrochloric acid (HCl, 10 mM) was added to the aqueous phase. The solvents were mixed in a separatory funnel at room temperature. After complete separation and distribution, the organic phase was used as the mobile phase and the aqueous phase as the stationary phase. During the fractionation, the CPC column was first filled with the stationary phase at a flow rate of 15 mL/min for 20 min at 500 rpm. Then the sample was prepared by dissolving 500 mg of crude extract in equal volumes of the mobile phase (without TEA) and the stationary phase (with HCl). Along with the injection of the extract, the mobile phase containing TEA was pumped through the column. The rotation speed increased to 1100 rpm, and the analysis was performed in displacement mode under ascending elution conditions. The mobile phase was delivered at a flow rate of 3 mL/min for the first 90 min, followed by 5 mL/min for the remaining 60 min. The run lasted 150 min; the first 90 min were obtained in the elution mode, which was followed by the extrusion mode for the following 60min. A total of 49 fractions were collected.

Based on the HPLC-MS fingerprints, the fractions were collected together based on their similarity. As a result, a total of 12 fractions were obtained, evaporated to dryness, and subjected to biological testing.

### 4.8. Equipment Used for the FAIA

The assay was performed in black 96-well CELLSTAR^®^ microplates (Greiner Bio-One, Kremsmünster, Austria). Fluorescence measurements were carried out using a GloMax^®^ Explorer Microplate Reader (Promega, Madison, WI, USA). The pH of the enzyme buffer was measured using a pH meter (Metler Toledo, Greifensee, Switzerland).

### 4.9. Preparing Samples for the FAIA Procedure

Acetylcholinesterase (AChE) from *Electrophorus electricus*, Type VI-S (lyophilized powder, Sigma-Aldrich, St. Louis, MO, USA) was dissolved in buffer to obtain a stock solution (50 U/mL). The enzyme solution used in the assay (3 U/mL) was prepared by diluting the stock solution with the appropriate amount of buffer. The assay buffer was prepared by dissolving Trizma base and bovine serum albumin (BSA), both purchased from Sigma-Aldrich (St. Louis, MO, USA), and adjusting the pH to 7.8. A 4-methylumbelliferyl acetate (4-MUA) solution (1.5 mg/mL) (Sigma-Aldrich, St. Louis, MO, USA) was obtained by dissolving the substance in a mixture of ethanol and water (2:1, *v*/*v*).

The crude extract and CPC fractions obtained from *Berberis julianae* fruits were grouped into 12 fractions based on their chemical composition. The dried fractions were dissolved in dimethyl sulfoxide (DMSO) to prepare working solutions at a concentration of 10 mg/mL. The DMSO (Avantor, Gliwice, Poland) was selected as solvent for FAIA due to its low toxicity, excellent solubilizing properties, and negligible interference with enzyme activity. Standards of berberine chloride, palmatine chloride, and demethylenberberine (Sigma-Aldrich, St. Louis, MO, USA), magnocurarine (ChemFaces, Wuhan, China), and jatrorrhizine (TRC Canada, North York, ON, Canada) were dissolved in dimethyl sulfoxide (DMSO) as working solutions at a concentration of 1 mg/mL.

### 4.10. FAIA Methodology

The AChE-inhibitory activity was determined using a 96-well black microplate (Whatman, Little Halfont, UK). Briefly, 10 μL of each test sample or DMSO (vehicle control) was mixed with 100 μL of the enzyme solution. The tested samples included the obtained fractions and individual alkaloid standards. Berberine (BER) was used as a positive control in the assay with fractions. All reaction mixtures were prepared in triplicate and incubated at 37 °C for 25 min. Subsequently, 10 μL of 4-methylumbelliferyl acetate (4-MUA) solution was added to each well, resulting in a final reaction volume of 120 μL, followed by an additional 20 min incubation at 37 °C. Based on the concentrations of the applied sample solutions (10 mg/mL for the fractions and 1 mg/mL for the individual alkaloid standards), the final concentrations in the assay wells were 0.833 mg/mL for the fractions and 0.0833 mg/mL for the individual alkaloid standards, respectively.

To account for the intrinsic fluorescence of the tested samples, sample-specific blank wells were prepared for each available fraction using fraction solutions at 5 mg/mL, (the results obtained at 10 mg/mL without background subtraction are presented in Figure S9 in the Supplementary Material). Corresponding blank measurements were also performed for DMB, BER, and DMSO. In the blank wells, the enzyme solution was replaced with the corresponding buffer while maintaining the same final volume as in the assay wells.

The fluorescence measured for each sample-specific blank was used as the background signal and subtracted from the fluorescence measured in the assay wells when calculating AChE inhibition. Vehicle background wells containing DMSO at the same final concentration as in the assay wells were also included. In addition, solvent control wells containing the same final concentration of DMSO but no test sample were used to account for any potential effect of DMSO on basal AChE activity. Fluorescence intensity was measured at an excitation wavelength of 365 nm and an emission wavelength of 450 nm. Sample-specific intrinsic fluorescence was assessed and accounted for using appropriate fluorescence blanks; however, potential quenching or enhancement of 4-methylumbelliferone (4-MU) fluorescence by the tested samples was not independently evaluated and therefore cannot be completely excluded. The percentage of AChE inhibition was calculated according to the following equation: % Inhibition = [(F_control_ − F_sample_)/F_control_] × 100, where F_control_ represents the fluorescence intensity of the control reaction containing DMSO and F_sample_ represents the fluorescence intensity measured in the presence of the tested sample. For background correction and calculation of AChE inhibition, the following equation was used: % Inhibition = [1 − (F_sample_ − F_sample blank_)/(F_control_ − F_control blank_)] × 100, where F_sample represents_ the fluorescence intensity measured in the presence of the tested sample, F_sample blank_ represents the corresponding sample-specific background fluorescence, F_control_ represents the fluorescence intensity of the DMSO control, and F_control blank_ represents the corresponding DMSO background fluorescence.

### 4.11. SH-SY5Y Cell Culture, Differentiation, and Cytotoxicity Assessment

Human neuroblastoma SH-SY5Y cells (ATCC CRL-2266, LGC Standards, Łomianki, Poland) were routinely maintained in DMEM/F-12 (Corning, NY, USA) supplemented with 10% fetal bovine serum (FBS, PAN-Biotech, Aidenbach, Germany) and 0.1% penicillin/streptomycin (Sigma-Aldrich, St. Louis, MO, USA) at 37 °C in a humidified atmosphere of 5% CO_2_.

For neuronal differentiation, cells were seeded onto 24-well plates or 60 mm culture dishes at a density of 3 × 10^4^ cells per well in 1 mL or 1.5 × 10^6^ cells per dish in 4 mL of 10% DMEM/F-12 supplemented with 0.1% penicillin/streptomycin (Day 0). On Day 1, the maintenance medium was replaced with differentiation medium consisting of DMEM/F-12 supplemented with 1% FBS HI, 10 μM all-trans retinoic acid (RA; Sigma-Aldrich, St. Louis, MO, USA), and 0.1% penicillin/streptomycin. The differentiation medium was renewed every 3 days. Cells were maintained under differentiation conditions for 14 days to obtain a mature neuronal phenotype, as previously described [54].

The efficiency of the neuronal differentiation protocol was verified by Western blot analysis, as previously described [55]. Briefly, on days 0 and 14 of differentiation, the culture medium was removed, and the cells were washed twice with ice-cold PBS. The cells were then scraped in radioimmunoprecipitation assay (RIPA) buffer and stored at −80 °C until analysis. On the day of analysis, the protein concentration of each sample was determined using the bicinchoninic acid (BCA) assay [56] and normalized across all samples. Equal amounts of protein (50 µg) were separated by electrophoresis on 10% SDS-PAGE gels (120 V, 1.5 h) and transferred onto 0.45 µm PVDF membranes (35 V, 16 h, 4 °C). The membranes were blocked with 1% bovine serum albumin (BSA) in TBST for 1 h at room temperature and subsequently incubated overnight at 4 °C with one of the following primary antibodies: rabbit anti-NeuN (1:5000; ABClonal; cat. no. A19086), rabbit anti-TUBB3 (1:20,000; ABClonal; cat. no. A17913), or mouse anti-GAPDH (1:50,000; ABClonal; cat. no. AC033). Following three washes with TBST, the membranes were incubated for 1 h at room temperature with the appropriate horseradish peroxidase (HRP)-conjugated secondary antibody: anti-rabbit IgG (1:2000; Thermo Fisher Scientific (Waltham, MA, USA); cat. no. 31460) or anti-mouse IgG (1:2000; Thermo Fisher Scientific; cat. no. 31430). The membranes were then washed three times with TBST and once with TBS before chemiluminescent detection using the Li-COR c-DiGit blot scanner (Lincoln, NE, USA). Band intensities were quantified using GelQuant.NET software (accessed 19 March 2026). Relative protein expression was calculated after normalization to GAPDH, which served as the loading control. Representative uncropped Western blot images are provided in the Supplementary Material Figure S6.

On Day 14, the differentiation medium was aspirated, and test samples were applied at concentrations of 200, 100, and 50 μg/mL, prepared by serial dilution in 1% DMEM/F-12 (without RA and antibiotics; 500 μL/well). Untreated control wells received only 1% DMEM/F-12. Background fluorescence was assessed in cell-free wells treated identically with 1% DMEM/F-12. Cells were incubated with test compounds for 24 h.

Cell metabolic activity was evaluated on Day 15 using the resazurin (AlamarBlue) reduction assay. Before the assay, representative photomicrographs of each well were acquired. A resazurin stock solution (0.25 mg/mL in PBS) was added to each well at 50 μL (final concentration: 0.025 mg/mL). Plates were incubated for 3 h at 37 °C with 5% CO_2_, after which fluorescence was measured using a microplate reader (FilterMax F5, Molecular Devices, San Jose, CA, USA) at excitation/emission wavelengths of 535/595 nm.

Statistical significance among tested conditions was determined by one-way analysis of variance (ANOVA) followed by Tukey’s multiple comparisons post hoc test, or by an unpaired *t*-test for Western blot results, using GraphPad Prism 8.0 software (San Diego, CA, USA). Differences were considered statistically significant at *p* < 0.05. For the cytotoxicity assays (Figure 7 and Figure 8), which involve two crossed experimental factors (concentration and differentiation state), statistical significance was additionally assessed by two-way ANOVA with concentration and differentiation state as factors, including their interaction term, performed separately for each tested fraction or alkaloid. These analyses were performed on the mean value of the technical replicate wells within each of *n* = 3 independent biological experiments, rather than on pooled technical replicate wells, to avoid pseudoreplication in inferential statistics. Fraction 7 in differentiated cells was excluded from the two-way ANOVA because only one of three planned independent experiments yielded usable data for this condition (*n* = 1); the corresponding results are reported descriptively only (Section 2.7). Similarly, one of three independent experiments for jatrorrhizine in differentiated cells was excluded from the two-way ANOVA (*n* = 2) owing to a documented culture-specific technical failure (Section 2.8). The two-way ANOVA recalculation described above, prompted by the reviewers’ comments, was performed with the assistance of Claude (Anthropic), an AI assistant; all analyses were reviewed and verified by the authors.

To evaluate the cytotoxic potential of the tested samples, the resazurin reduction assay was performed on both undifferentiated and RA-differentiated SH-SY5Y cells. The two models were employed to distinguish between general cytotoxic and antiproliferative effects characteristic of actively dividing neuroblastoma cells and neuron-specific cytotoxicity observed in post-mitotic, differentiated cells with an established neuronal phenotype [57,58]. Since undifferentiated SH-SY5Y cells retain proliferative capacity, a reduction in metabolic activity in this model reflects a combined cytotoxic and cytostatic response. In contrast, differentiated cells arrested in the G0/G1 phase no longer divide; therefore, changes in resazurin reduction in this model are not confounded by cell proliferation and more directly reflect changes in cellular metabolic/reductase activity associated with loss of cell viability [59]. This dual-model approach enables a more comprehensive assessment of the biological activity of the tested samples and improves the translational relevance of the findings to neuronal physiology in vivo, as previously demonstrated for compounds whose toxicity varies with the differentiation status of the SH-SY5Y cell line [60].

**Figure 13:**
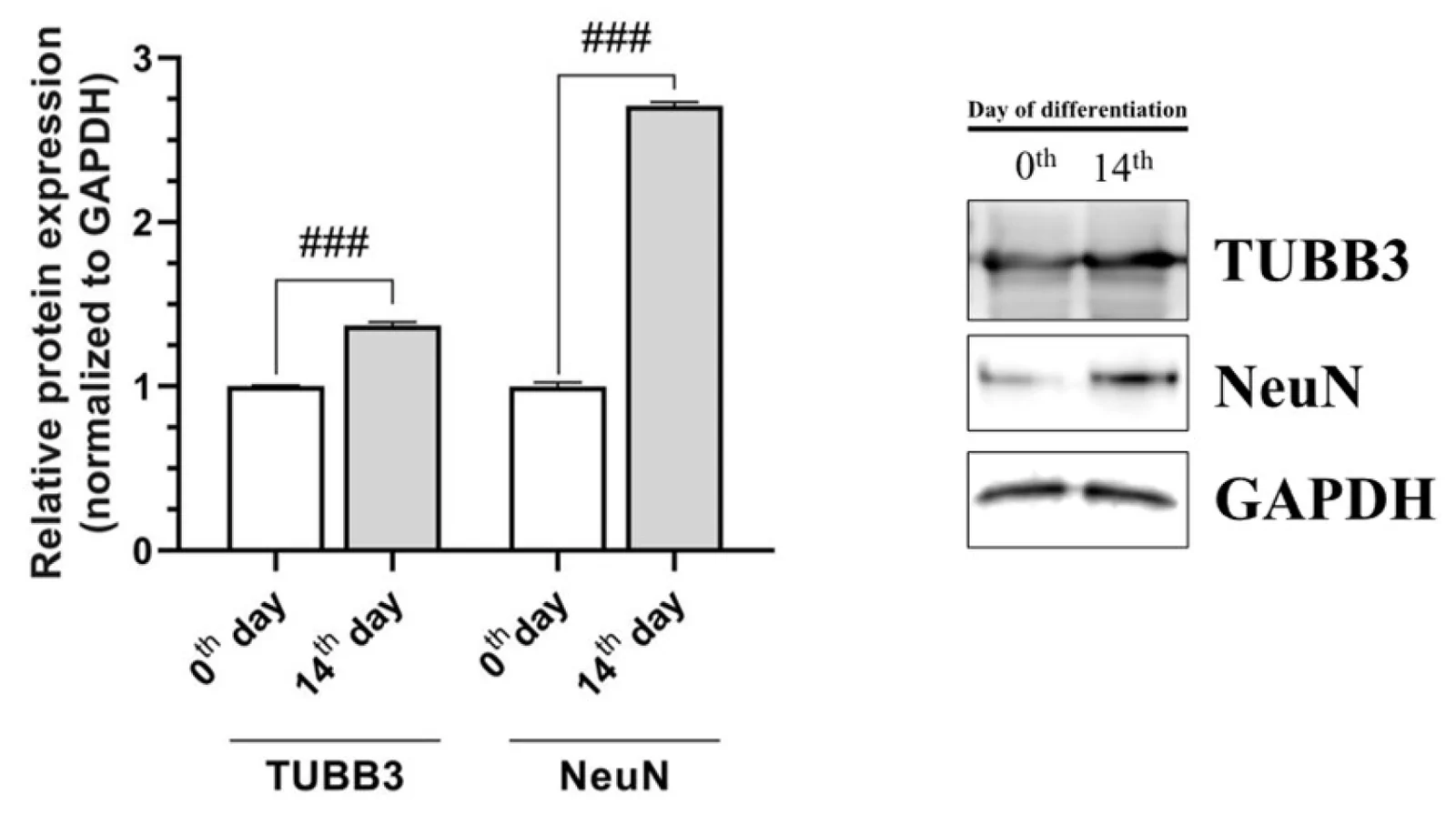
Efficiency of the neuronal differentiation protocol. Protein expression of TUBB3 and NeuN in SH-SY5Y cells at the beginning (day 0) and the end (day 14) of the differentiation protocol. Data are presented as the mean ± SD (*n* = 3). Statistical significance was determined using an unpaired *t*-test. ### *p* < 0.001 versus day 0.

To confirm the effectiveness of the neuronal differentiation protocol, the expression of two well-established neuronal markers, neuronal nuclei (NeuN) and class III β-tubulin (TUBB3), which are upregulated during neuronal differentiation, was evaluated [61]. After 14 days of differentiation, SH-SY5Y cells exhibited significant increases in the expression of both TUBB3 (37.17%) and NeuN (170.89%) compared with undifferentiated cells (Figure 13), confirming successful differentiation.

## 5. Conclusions

In this study, a novel microplate-based Fluorescence Acetylcholinesterase Inhibition Assay (FAIA) using 4-methylumbelliferone acetate (4-MUA) as a fluorogenic substrate was developed and successfully applied to screen acetylcholinesterase inhibitors in *Berberis julianae* fruit extracts and CPC fractions. Phytochemical analysis of the most active fraction revealed the presence of protoberberine alkaloids, including berberine, demethylberberine, jatrorrhizine, and palmatine, which are likely responsible for the observed AChE-inhibitory activity. In addition, the proposed MS/MS fragmentation pathways for these alkaloids enhance understanding of their fragmentation behavior and may facilitate the identification of related compounds in future phytochemical studies. This study also provides the first comparative evaluation of the cytotoxicity of protoberberine alkaloids and selected CPC fractions from *Berberis julianae* fruits in both differentiated and undifferentiated SH-SY5Y cells. All tested protoberberine alkaloids exhibited concentration-dependent cytotoxicity, while differentiated SH-SY5Y cells were generally more susceptible to their cytotoxic effects than undifferentiated cells. In contrast, the selected CPC fractions did not consistently show this pattern, suggesting that interactions among multiple phytochemical constituents may modulate their overall cytotoxicity. Quantitative integration of AChE inhibition and cytotoxicity further underscored the importance of considering biological activity alongside cellular safety. BER exhibited greater AChE-inhibitory potency than DMB, with IC_50_ values of 47.51 and 64.44 µg/mL, respectively. However, DMB showed a more favorable selectivity margin, particularly in undifferentiated SH-SY5Y cells (SI ≈ 2.3), compared with BER (SI ≈ 1.4). In differentiated cells, the selectivity margins were considerably narrower (SI ≈ 1.1 for DMB and <1.05 for BER), indicating that concentrations associated with substantial AChE inhibition were close to those affecting cell viability. These findings highlight that AChE-inhibitory potency alone is insufficient to evaluate the potential of protoberberine alkaloids as neuroactive compounds and should be considered alongside their cytotoxicity.

Overall, the presented workflow combining bioactivity-guided fractionation, phytochemical characterization, AChE inhibition screening, and cellular safety assessment may be applied not only to *Berberis* spp. but also to other alkaloid-rich medicinal plants requiring rapid identification and evaluation of biologically active constituents.

## 6. Patents

The results presented in this study are related to the Polish patent application No. P.455145, entitled “A fluorometric method for the detection of acetylcholinesterase inhibitors” (published on 19 March 2026).

## Figures and Tables

**Table 2:** Biphasic solvent systems evaluated for CPC fractionation of the *Berberis julianae* fruit extract (the selected solvent system is presented in bold).

№	Solvent System	Volume Ratio (*v*/*v*)	Modifiers
1	methyl *tert*-butyl ether: water	1:1	10 mM TEA 10 mM HCl
**2**	**methyl *tert*-butyl ether: butanol: acetonitrile: water**	**2:2:1:5**	**10 mM TEA** **10 mM HCl**
3	butanol: acetonitrile: water	10:2:8	0.5% TEA
4	methyl *tert*-butyl ether: acetonitrile: water	2:2:3	10 mM TEA 10 mM HCl
5	chloroform: methanol: water	2:2:3	0.1 M HCl
6	butanol: hexane: acetonitrile: water	8:2:5:13	0.3% HCl
7	chloroform: methanol: water	5:4:4	0.5% CH_3_COOH

Legend: TEA = triethylamine; HCl = hydrochloric acid. Solvent systems were evaluated for their suitability in the fractionation of isoquinoline alkaloids. The selected solvent system is indicated in bold.

## Data Availability

All data are included in the manuscript and Supplementary File.

## Supplementary Materials

The following supporting information can be downloaded at https://www.mdpi.com/article/10.3390/molecules31183182/s1, Figure S1. Effect of blank subtraction on the calculated inhibitory activity of berberine (BER) against AChE at different working concentrations (0.001, 0.01, 0.1, 1). Orange curves indicate uncorrected data, whereas blue curves indicate blank-corrected data. Figure S2. Acetylcholinesterase (AChE) inhibitory activity of fraction 7 (the active fraction) obtained by CPC from the *B. julianae* fruit extract, evaluated at two working concentrations (5 and 2 mg/mL). Berberine (BER, 1 mg/mL) was used as the reference standard. Error bars represent the standard deviation (SD) of three independent measurements (*n* = 3). Table S1. Partition coefficients (*K*) of representative chromatographic peaks determined during solvent system screening for countercurrent partition chromatography (CPC). Figure S3. UHPLC–ESI–QTOF–MS base peak chromatograms (BPCs) of *Berberis julianae* fruit crude extract acquired in ESI+ (top) and ESI− (bottom) modes. The identified compounds are annotated in Table 2 (manuscript). Figure S4. MS/MS spectra of identified and unidentified compounds detected in *Berberis julianae* fruit crude extract by UHPLC–ESI–QTOF–MS/MS. Figure S5. Countercurrent chromatogram of *Bereberis julianae* fruit extract obtained by centrifugal partition chromatography (CPC). Figure S6. Representative uncropped Western blot images of TUBB3, NeuN, and GAPDH. The corresponding bands are indicated by red boxes. Figure S7. Proposed MS/MS fragmentation pathways of the protonated ions of magnocurarine (MGC), demethyleneberberine (DMB), jatrorrhizine (JAT), berberine (BER), and palmatine (PAL) acquired in positive ESI mode. Figure S8. Base peak chromatograms (BPCs) of CPC fractions 6, 7, and 10 selected for cytotoxicity evaluation in differentiated and undifferentiated SH-SY5Y cells using the Alamar Blue reduction assay. Major alkaloids tentatively identified by HPLC-ESI-QTOF-MS/MS, indicated and listed in Table 2 (manuscript). Figure S9. AChE-inhibitory activity of CPC fractions tested at a working concentration of 10 mg/mL with no correction for sample-specific background fluorescence. Berberine (BER) and DMSO were included as controls. Error bars represent the standard deviation (SD) of three independent measurements (*n* = 3). Figure S10. Proposed MS/MS fragmentation pathways of the identified protoberberine alkaloids in active fraction Fr 7: (A) berberine, (B) jatrorrhizine, and (C) palmatine.

## Abbreviations

The following abbreviations are used in this manuscript:

ACh	Acetylcholine
AChE	Acetylcholinesterase
AD	Alzheimer’s Disease
FAIA	Fluorescence Acetylcholinesterase Inhibition Assay
HPLC	High-Performance Liquid Chromatography
LC-MS	Liquid Chromatography–Mass Spectrometry
TLC	Thin-Layer Chromatography
CPC	Centrifugal Partition Chromatography
pH-ZRP CPC	pH-Zone Refining Centrifugal Partition Chromatography
HCl	Hydrochloric Acid
TEA	Trimethylamine
BER	Berberine
GAL	Galantamine
4-MUA	4-Methylumbelliferone acetate
DMSO	Dimethylsulfoxide
FBS	Fast Blue Salt
DTNB	5,5′-dithiobis(2-nitrobenzoic acid)
MGC	Magnocurarine
DMB	Demethyleneberberine
JAT	Jatrorrhizine
PAL	Palmatine
IC_50_	Half-Maximal Inhibitory Concentration
