# Supplementary material for: A Fluorescence-Based Acetylcholinesterase Inhibition Assay for Bioactivity-Guided Identification of Neuroactive Protoberberine Alkaloids from Berberis julianae C.K. Schneid
Source: Molecules. 2026 Sep 10;31(18):3182. doi: 10.3390/molecules31183182 (PMC13609244; doi:10.3390/molecules31183182)
Supplement: Supplementary file 1 [file molecules-31-03182-s001.zip › molecules-4500635-supplementary.pdf]

Supplementary Materials for

«A Fluorescence-Based Acetylcholinesterase Inhibition Assay for Bioactivity-Guided Identification of Neuroactive Protoberberine Alkaloids from *Berberis julianae* C.K. Schneid.»

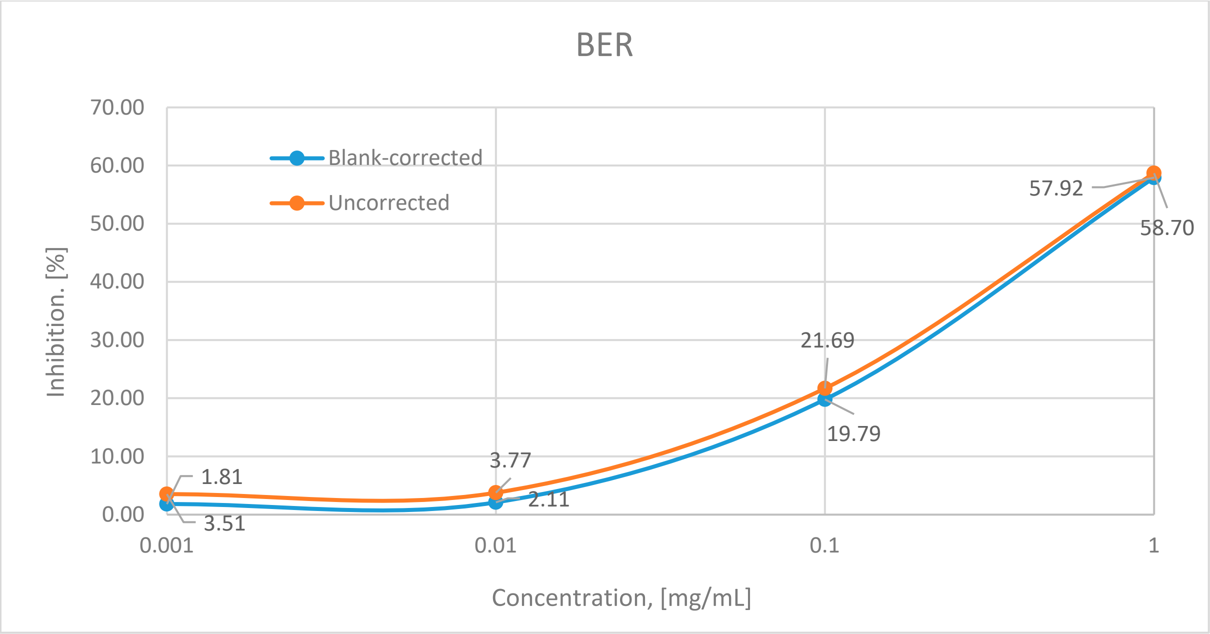

Figure S1. Effect of blank subtraction on the calculated inhibitory activity of berberine (BER) against AChE at different working concentrations (0.001, 0.01, 0.1, 1). Orange curves indicate uncorrected data, whereas blue curves indicate blank-corrected data.

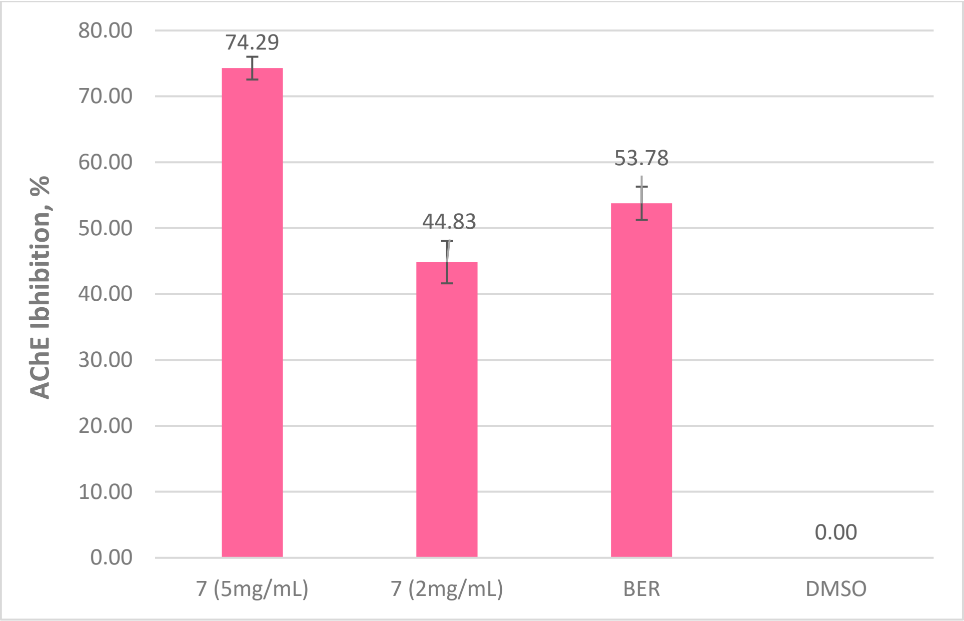

Figure S2. Acetylcholinesterase (AChE) inhibitory activity of fraction 7 (the active fraction) obtained by CPC from the *B. julianae* fruit extract, evaluated at two working concentrations (5 and 2 mg/mL). Berberine (BER, 1 mg/mL) was used as the reference standard. Error bars represent the standard deviation (SD) of three independent measurements (n = 3).

Table S1. Partition coefficients (*K*) of representative chromatographic peaks determined during solvent system screening for countercurrent partition chromatography (CPC).

	neg				pos			
sol.system	rt time	upper	lower	k	rt time	upper	lower	k
1	12.858	4975162.91	91532497.11	0.054354	2.915	7542834.75	265324499.4	0.028429
	14.526	3830899.43	47669285.17	0.080364	5.74	2073307.93	35103541.47	0.059063
	15.86	6606975.53	11014092.56	0.599866	17.989	2312102.58	84154757.1	0.027474
	23.532	158713121.1	30162683.76	5.261903	23.152	114511906.7	423410967.4	0.270451
	21.364	5751344.98	4056048.22	1.417968	25.984	1182018.6	38042139.08	0.031071
					26.734	27569031.13	91817342.43	0.30026
					21.986	19300005.33	9952587.66	1.939195
2	2.349	19858160.57	183383694.9	0.108287	1.761	3431336.93	3628275.26	0.945721
	4.768	329964.84	9903916.38	0.033317	2.344	16369649.75	18920073.28	0.8652
	12.94	8233408.71	61425041.46	0.13404	2.927	179385020.9	166339312.3	1.078428
	16.525	460254.25	13623785.55	0.033783	12.759	9494596.42	2534455.96	3.746207
	18.027	6990683.95	4510003.53	1.55004	13.926	14308169.8	3330525.59	4.29607
	17.276	1579531.22	4510003.53	0.350228	14.676	17771508.15	16594435.04	1.070932
	18.777	248878.93	2506401.05	0.099297	15.009	19694750.97	18759219.94	1.04987
	21.946	1284550.55	1856252.85	0.692013	15.759	7439933.15	4820227.2	1.543482
					19.927	41822639.96	12607029.2	3.317406
					17.51	8401247.76	10755257.96	0.781129
					23.177	320084511	147974883.7	2.1631
					26.678	98044076.19	27938989.3	3.509221
					18.01	25295772.39	8525133.81	2.967199
3	2.338	19840624.57	183383694.9	0.108192	2.594	145909868.3	752834342.2	0.193814
	12.928	5069166.03	61425041.46	0.082526	10.006	40076110.59	11150048.61	3.594254
	14.512	620634.25	13623785.55	0.045555	17.584	1846280.23	20072494.46	0.091981
	22.101	1603111.64	1856252.85	0.863628	15.085	3542934.45	45239473.91	0.078315
	18.766	204392.16	2506401.05	0.081548	19.916	4753701.1	11062887.95	0.429698
	22.1	1603111.64	1856252.85	0.863628	24.25	37316472.9	276201163.7	0.135106
	18.015	308869.13	2573578.32	0.120015				
4	2.335	22955678.68	93479140.98	0.24557	1.76	6515450.25	2627004.94	2.480182
	12.925	16770600.4	988789.18	16.96074	2.26	21395311.87	91063526.52	0.234949
	15.927	975233.23	1798818.59	0.542152	3.093	89501396.5	518702896.6	0.172548
	17.429	20819125.44	3373315.59	6.17171	13.001	6898229.32	5978199.57	1.153897

	18.012	13532611.79	2431234.7	5.566148	20.579	120111755.3	14400737.38	14400737
	20.597	35352878.01	4384064.45	8.06395	19.747	35882879.82	7586564.98	14400737
	23.183	4958099.91	1521258.05	3.25921	22.414	3595082.94	30172616.05	14400737
5	18.021	896304.6	34286763.7	0.026141	2.425	91063526.52	21395311.87	4.256237
	20.106	515455.11	4083555.66	0.126227	3.258	89501396.5	518702896.6	0.172548
	20.606	7509989.13	49225283.19	0.152564	17.331	117351405.1	13251769.03	8.855528
					17.998	95422545.33	11050173.11	8.635389
					19.747	35882879.82	7586564.98	4.729793
					20.579	120111755.3	14400737.38	8.340667
					22.495	24192215.93	30172616.05	0.801794
					23.494	65950986.37	14569187.66	4.526744
					24.161	121834687	101811941.9	1.196664
					13.001	6898229.32	5978199.57	1.153897
					14.833	1839810.91	36563465.63	0.050318
					15.166	4467315.43	30139057.67	0.148223
6	2.264	11664148.73	73662093.83	0.158347	1.75	1249735.83	9442827.41	0.132348
	12.855	5538933.72	1099514.95	5.037616	2.416	8912907.62	40666598.52	0.21917
	14.439	376449.92	13889632.82	0.027103	2.999	27110157.51	16537857.88	1.639279
	20.027	659765.62	2024517.35	0.325888	12.909	1446666.88	9734968.4	0.148605
					14.491	4235364.08	51007448.66	0.083034
					15.074	3222799.38	46951339.96	0.068641
					15.74	707794.74	10808324	0.065486
					19.821	8179978.4	23224329.11	0.352216
					20.654	9438292.88	18620822.97	0.506868
					21.32	297196.13	5553481.72	0.053515
					23.735	20852958.33	79992599.57	0.260686
					24.152	12514403.76	66217556.46	0.188989
					24.901	7172236.53	70358831.65	0.101938
					25.485	11259505.33	59691607.3	0.188628
7	2.348	145255143.8	2349157.88	61.83286	2.337	65804464.22	6890095.35	9.550588
	12.355	4027312.1	521581.47	7.721348	3.42	10093055.23	39314780	0.256724
	15.857	5088244.49	1387902.1	3.666141	15.745	31196047.06	19907303.06	1.567065
	17.525	8359605.41	637373.88	13.1157	17.494	34281103.57	24468434.93	1.401034
	18.109	8829999.48	1778754.97	4.964146	17.994	21966930.16	106251154.5	0.206745
					19.909	26800815.89	104334687.5	0.256873

20.576	32062549.58	24741621.17	1.295895
21.825	11586898.15	25743990.58	0.450082
23.907	118070503.5	145418260.4	0.811937
24.906	195691365.5	34483093.33	5.674995

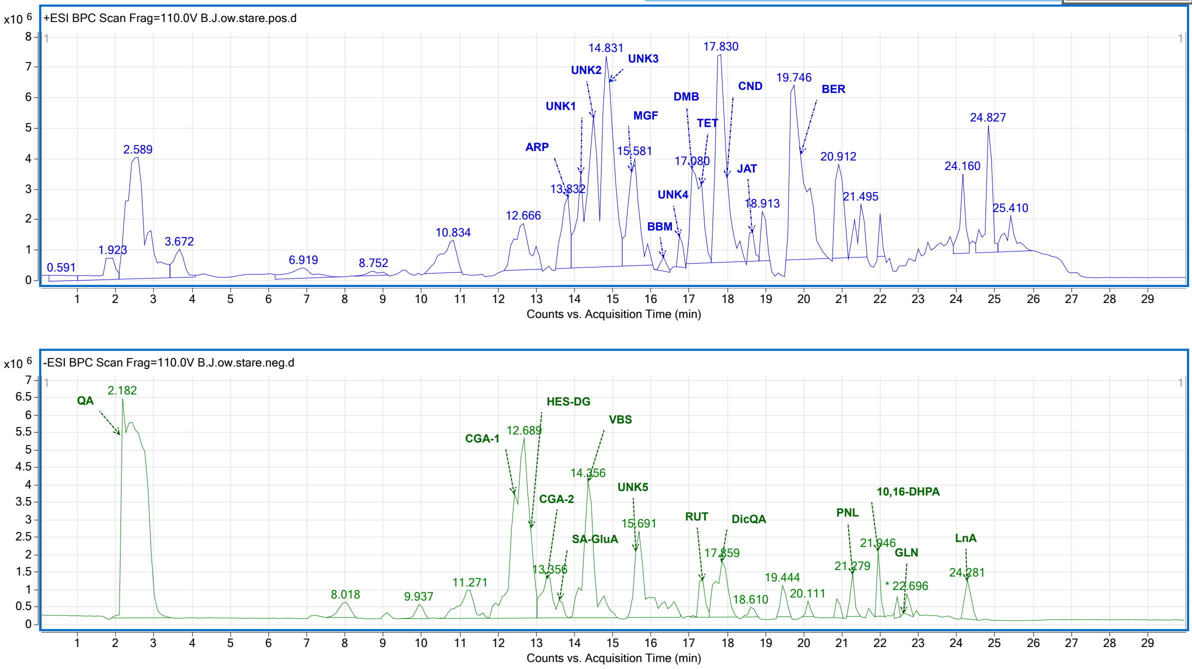

Figure S3. UHPLC–ESI–QTOF–MS base peak chromatograms (BPCs) of *Berberis julianae* fruit crude extract acquired in ESI+ (top) and ESI– (bottom) modes. The identified compounds are annotated in Table 2 (manuscript).

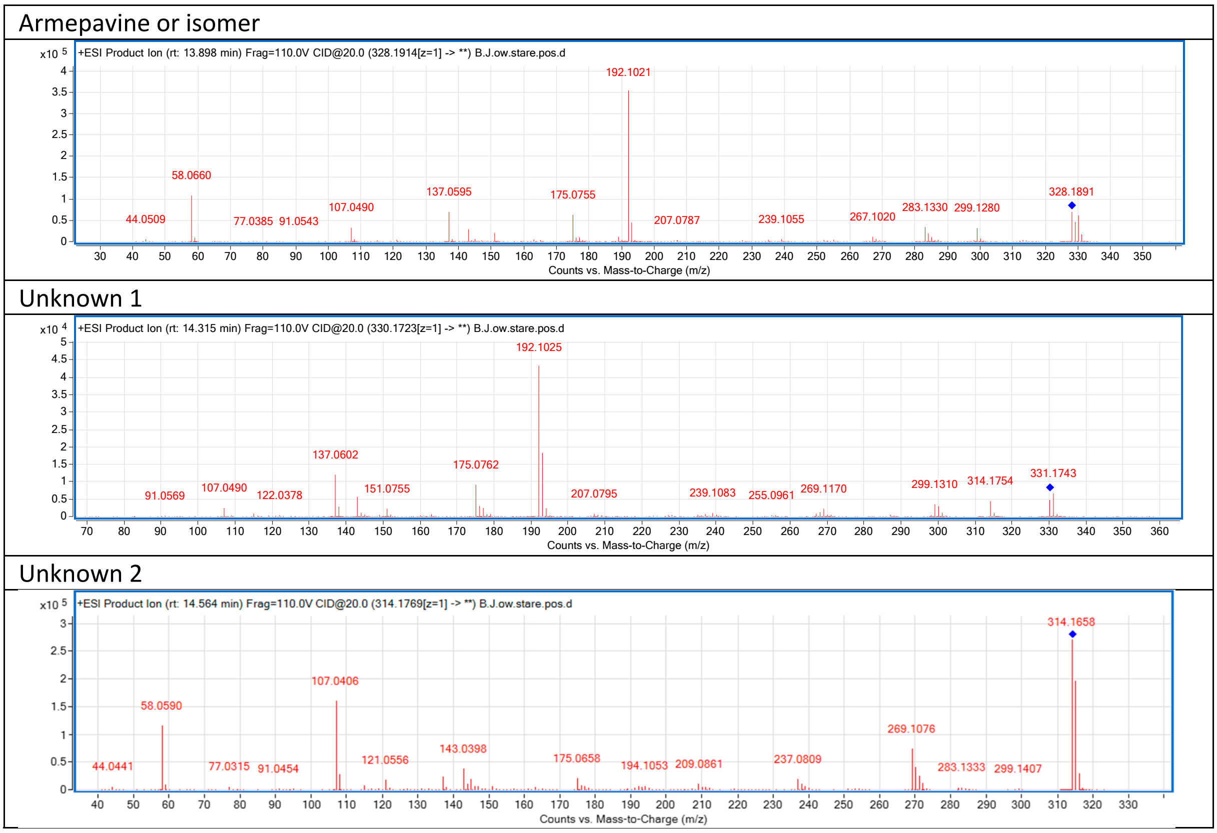

Unknown 3

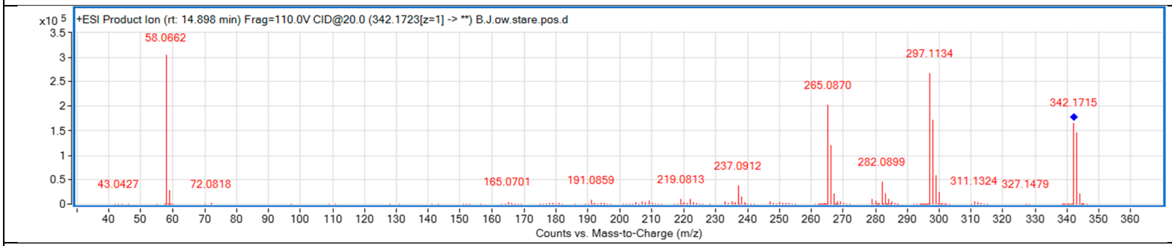

Magnoflorine

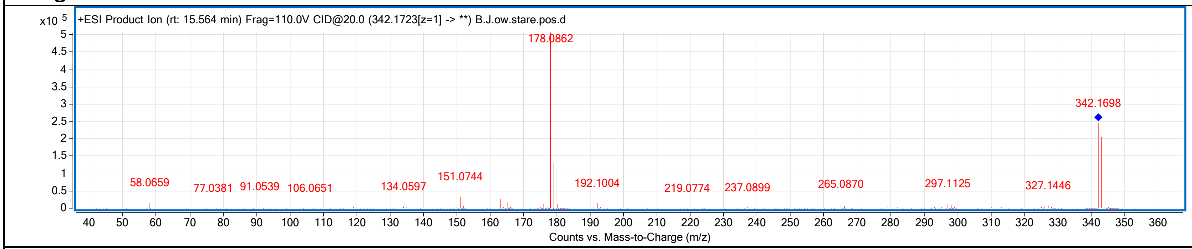

Berbamine or isomer

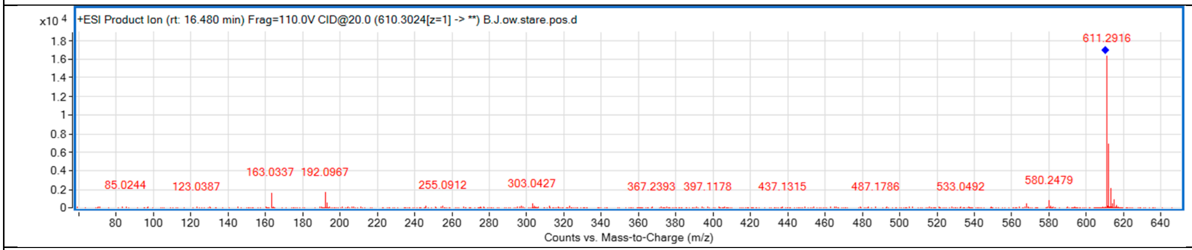

Unknown 4

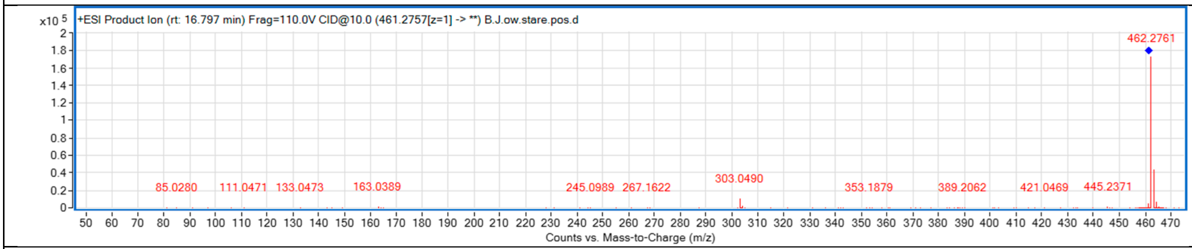

Demethyleneberberine

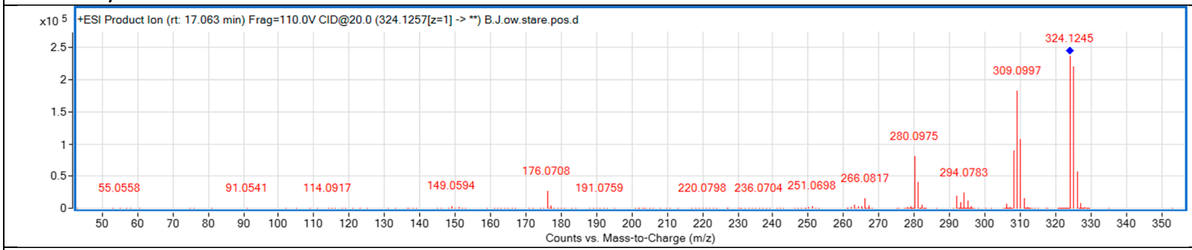

Tetrandrine or isomer

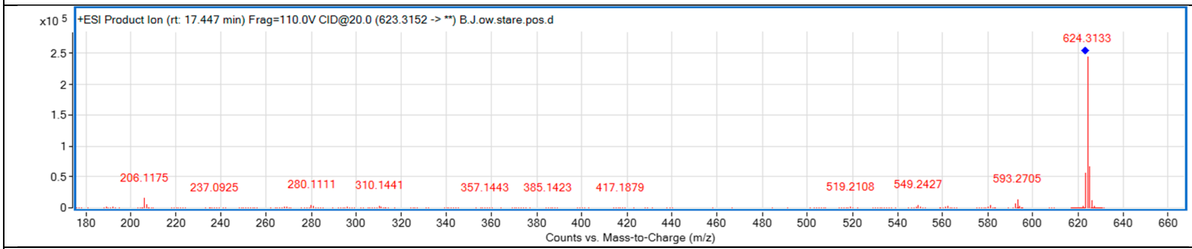

Canadine

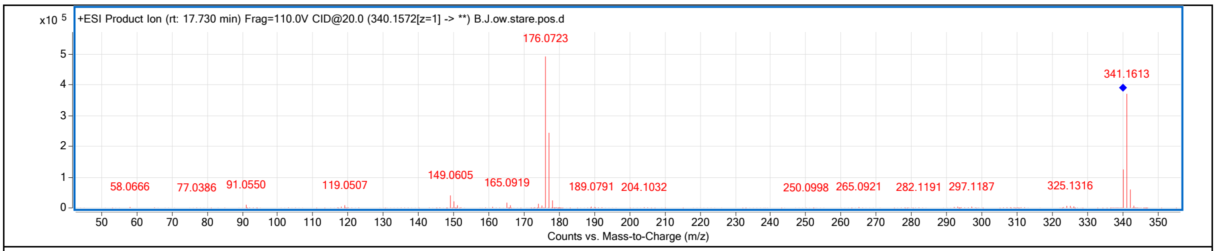

Jatrorrhizine

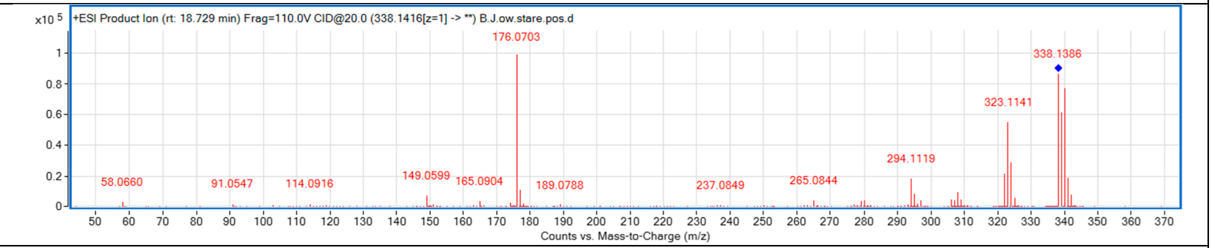

Berberine

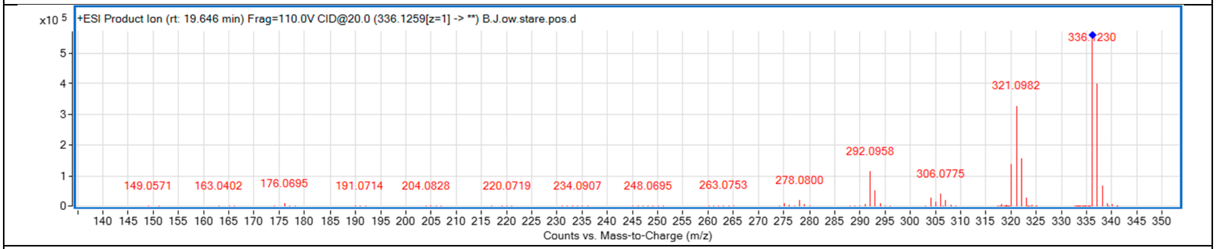

Quinic acid

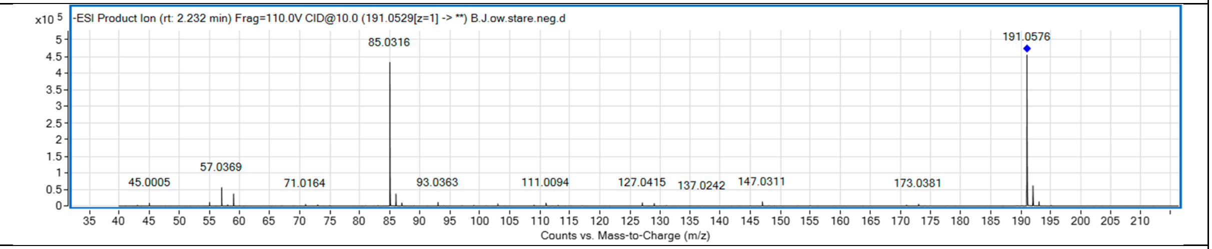

Chlorogenic acid 1

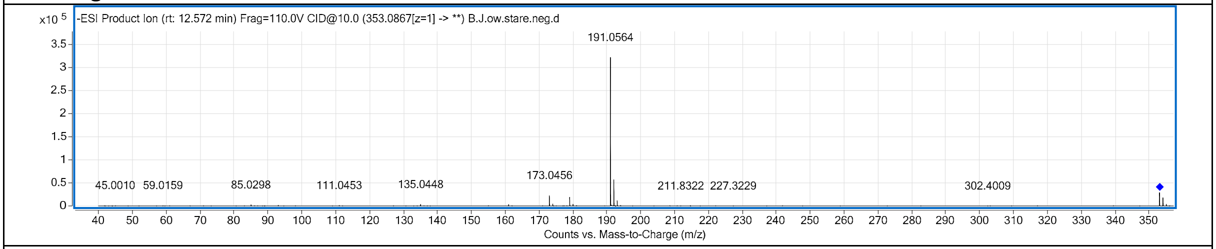

Chlorogenic acid 2

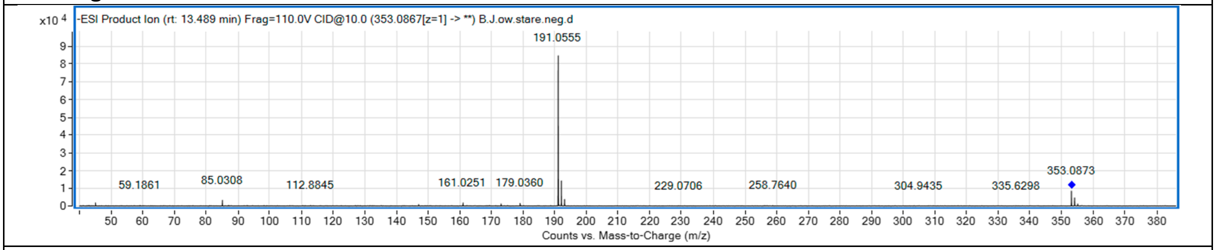

Sinapinic acid-*O*-glucuronide or isomer

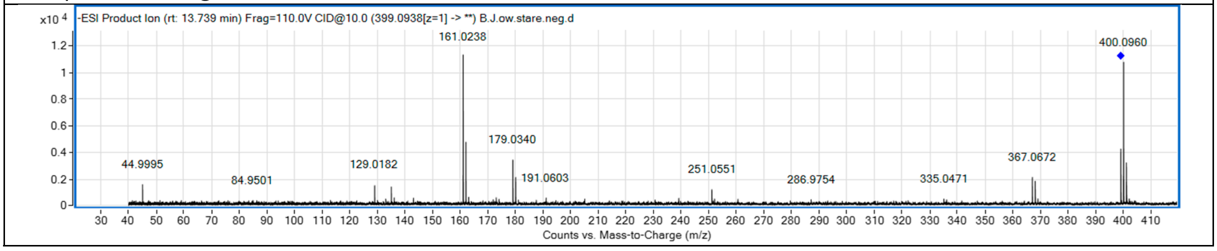

Verbasoside

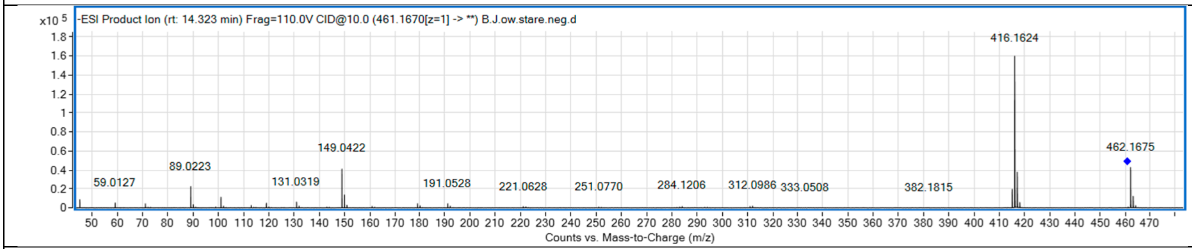

Unknown 5

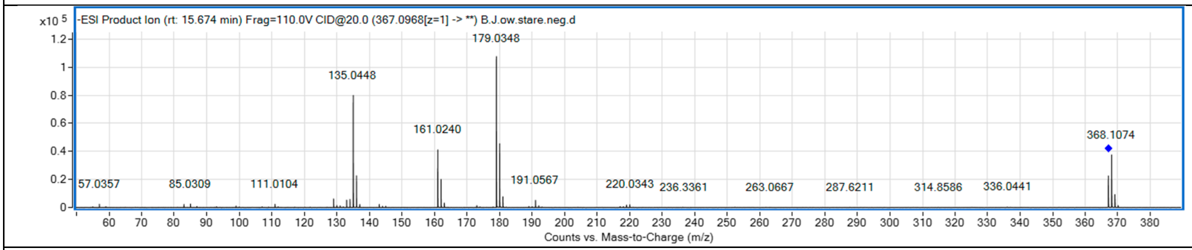

Rutin (quercetin-3-*O*-rutinoside)

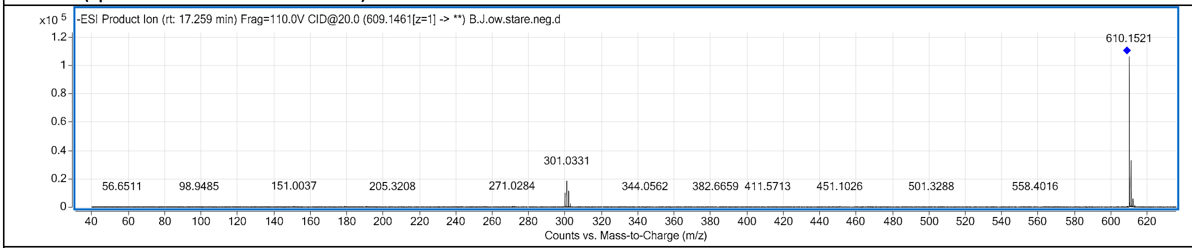

Dicaffeoylquinic acid

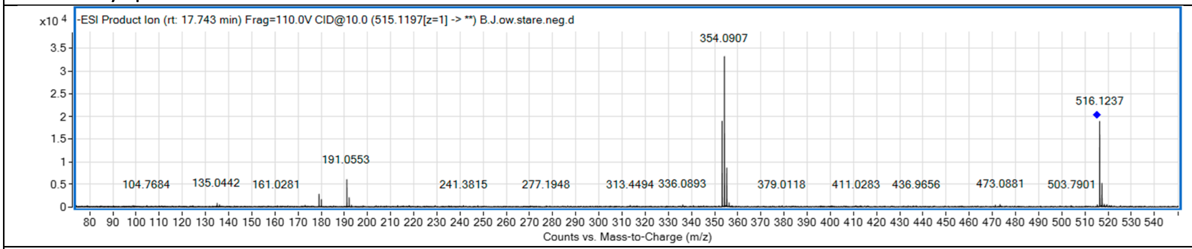

Hesperetin diglucuronide or isomer

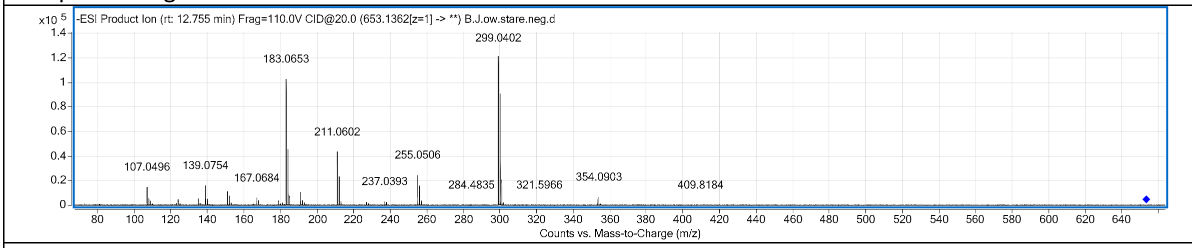

Pinellic acid

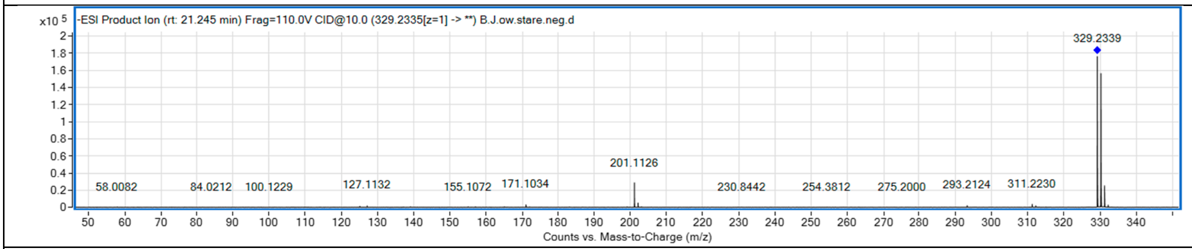

10,16-Dihydroxyhexadecanoic acid

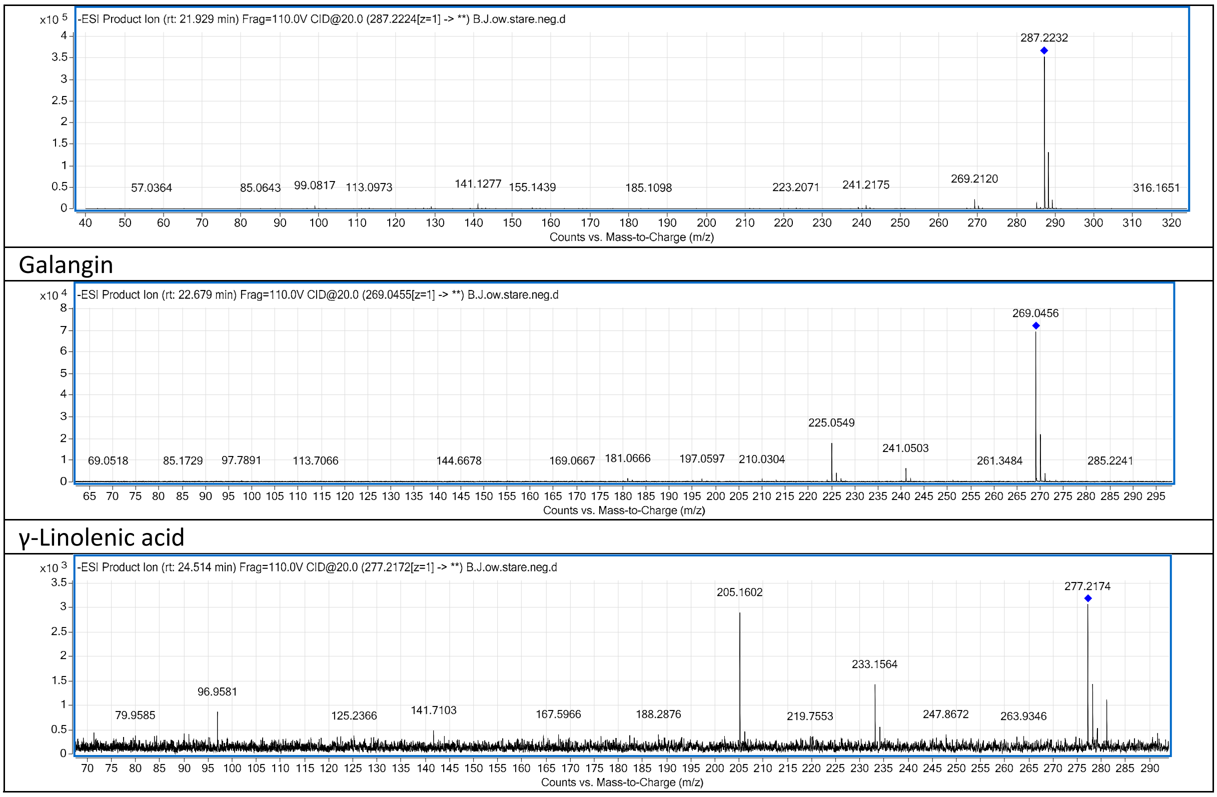

Figure S4. MS/MS spectra of identified and unidentified compounds detected in *Berberis julianae* fruit crude extract by UHPLC–ESI–QTOF–MS/MS.

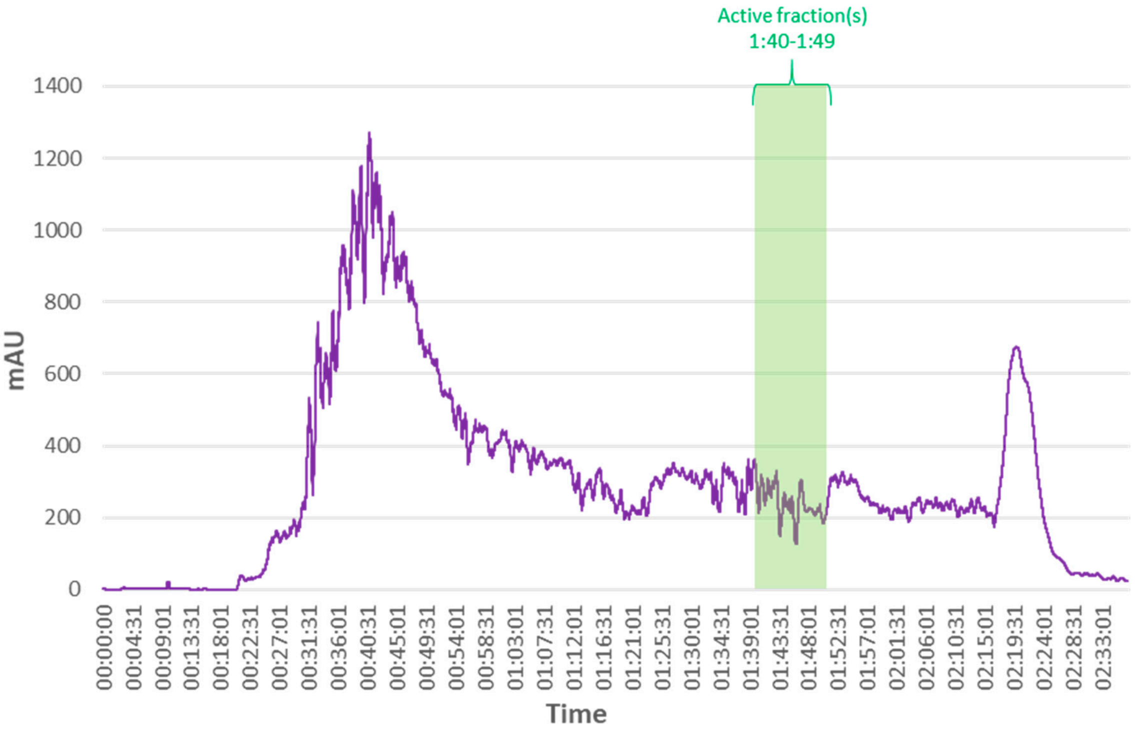

Figure S5. Countercurrent chromatogram of *Bereberis julianae* fruits extract obtained by centrifugal partition chromatography (CPC).

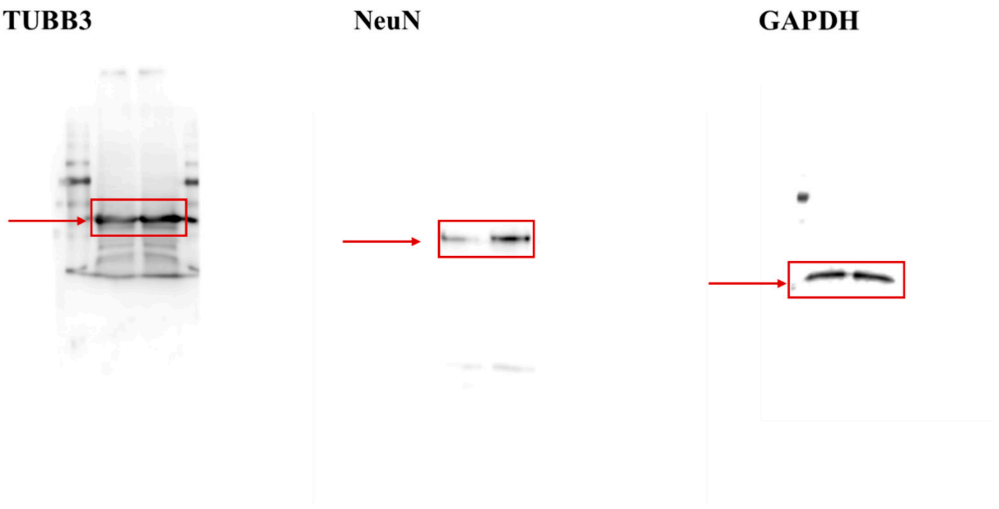

Figure S6. Representative uncropped Western blot images of TUBB3, NeuN, and GAPDH. The corresponding bands are indicated by red boxes.

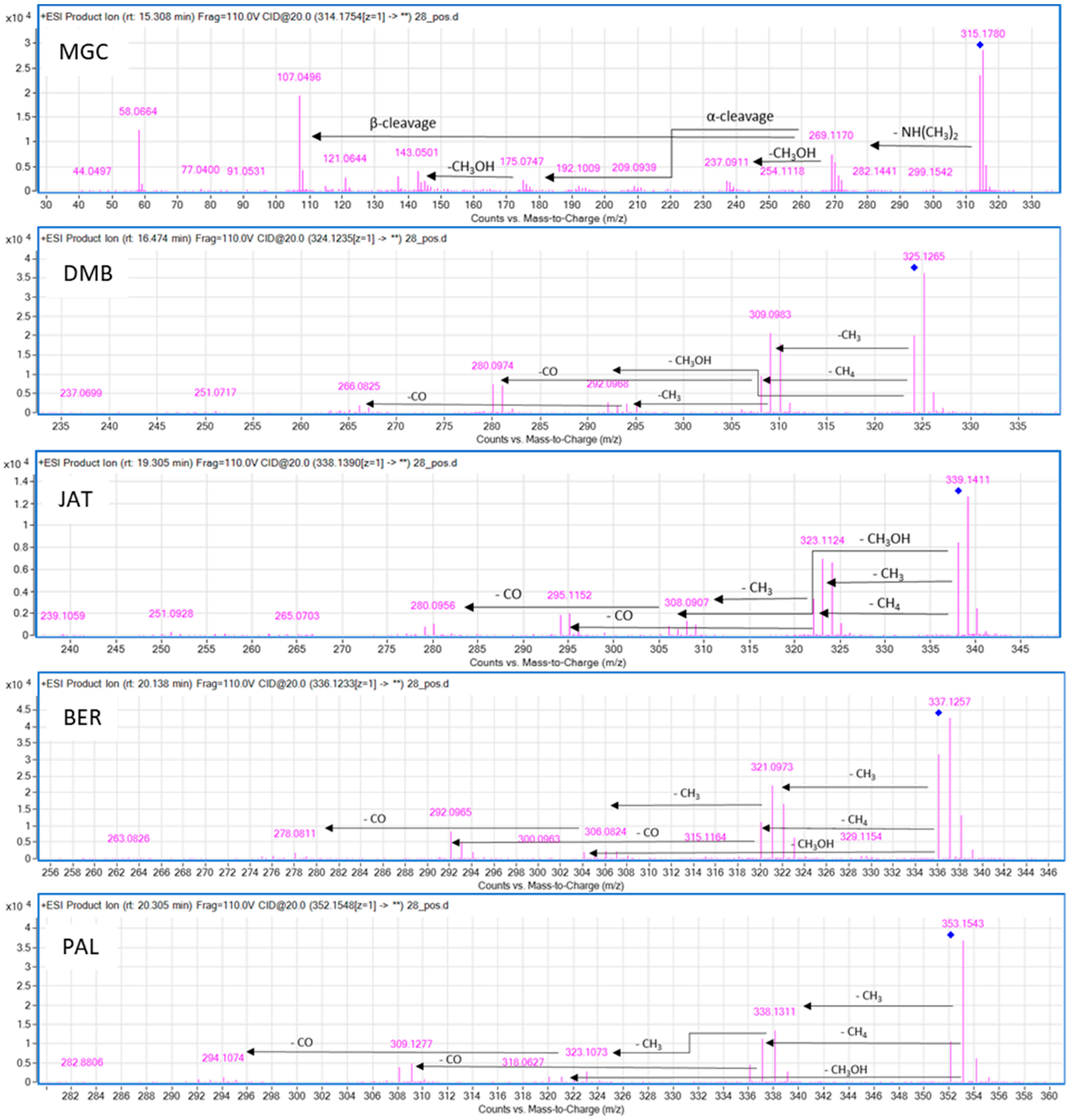

Figure S7. Proposed MS/MS fragmentation pathways of the protonated ions of magnocurarine (MGC), demethyleneberberine (DMB), jatrorrhizine (JAT), berberine (BER), and palmatine (PAL) acquired in positive ESI mode.

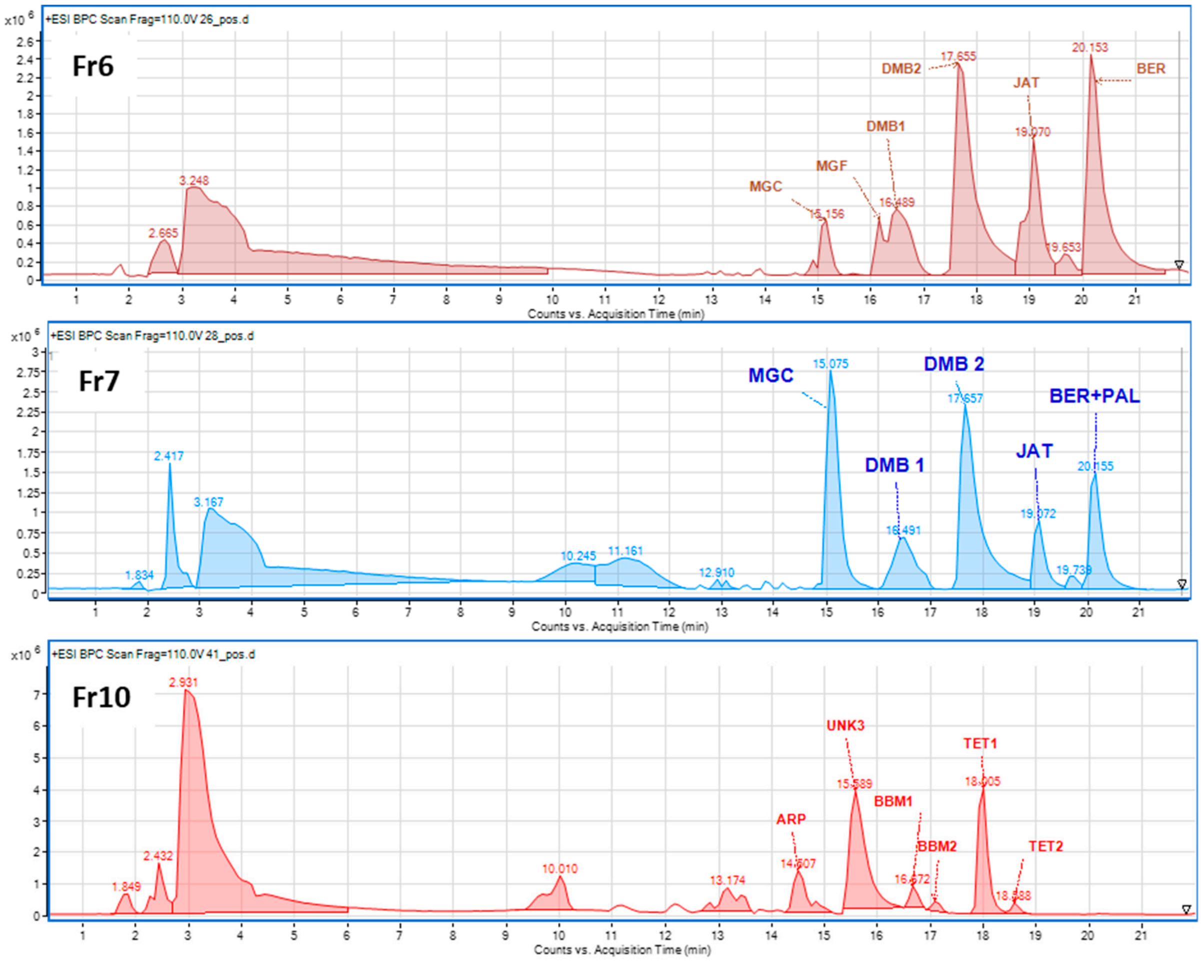

Figure S8. Base peak chromatograms (BPCs) of CPC fractions 6, 7, and 10 selected for cytotoxicity evaluation in differentiated and undifferentiated SH-SY5Y cells using the Alamar Blue reduction assay. Major alkaloids tentatively identified by HPLC-ESI-QTOF-MS/MS, indicated and listed in Table 2 (manuscript).

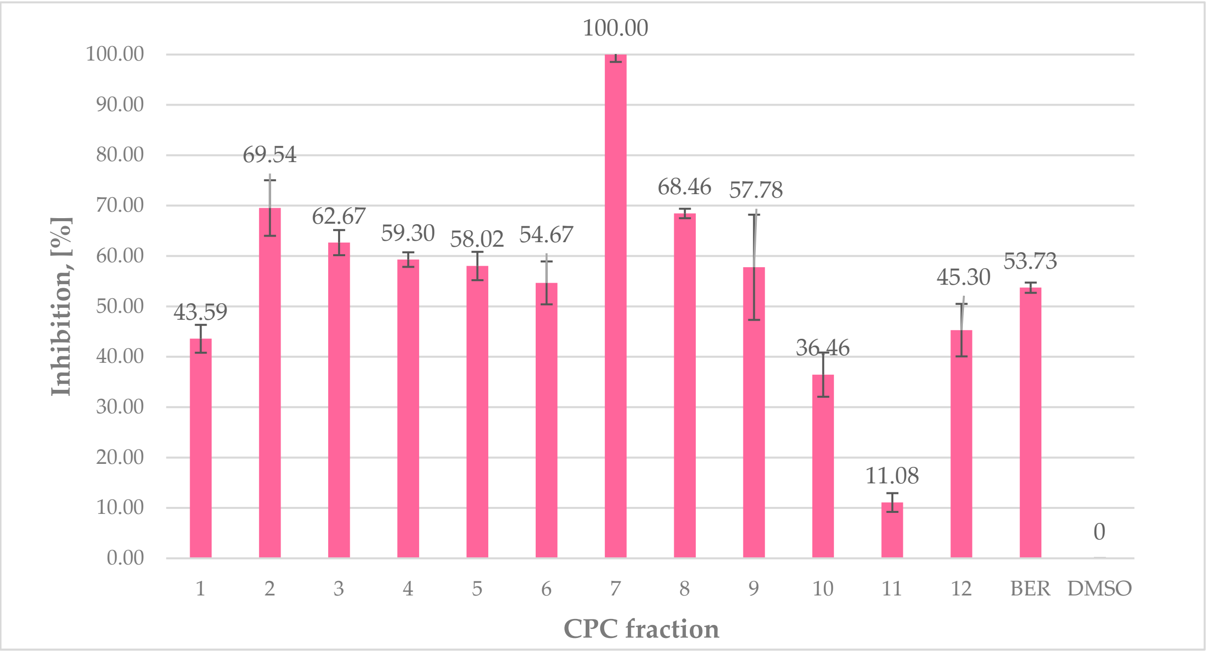

Figure S9. AChE inhibitory activity of CPC fractions tested at a working concentration of 10 mg/mL with no correction for sample-specific background fluorescence. Berberine (BER) and DMSO were included as controls. Error bars represent the standard deviation (SD) of three independent measurements (n = 3).

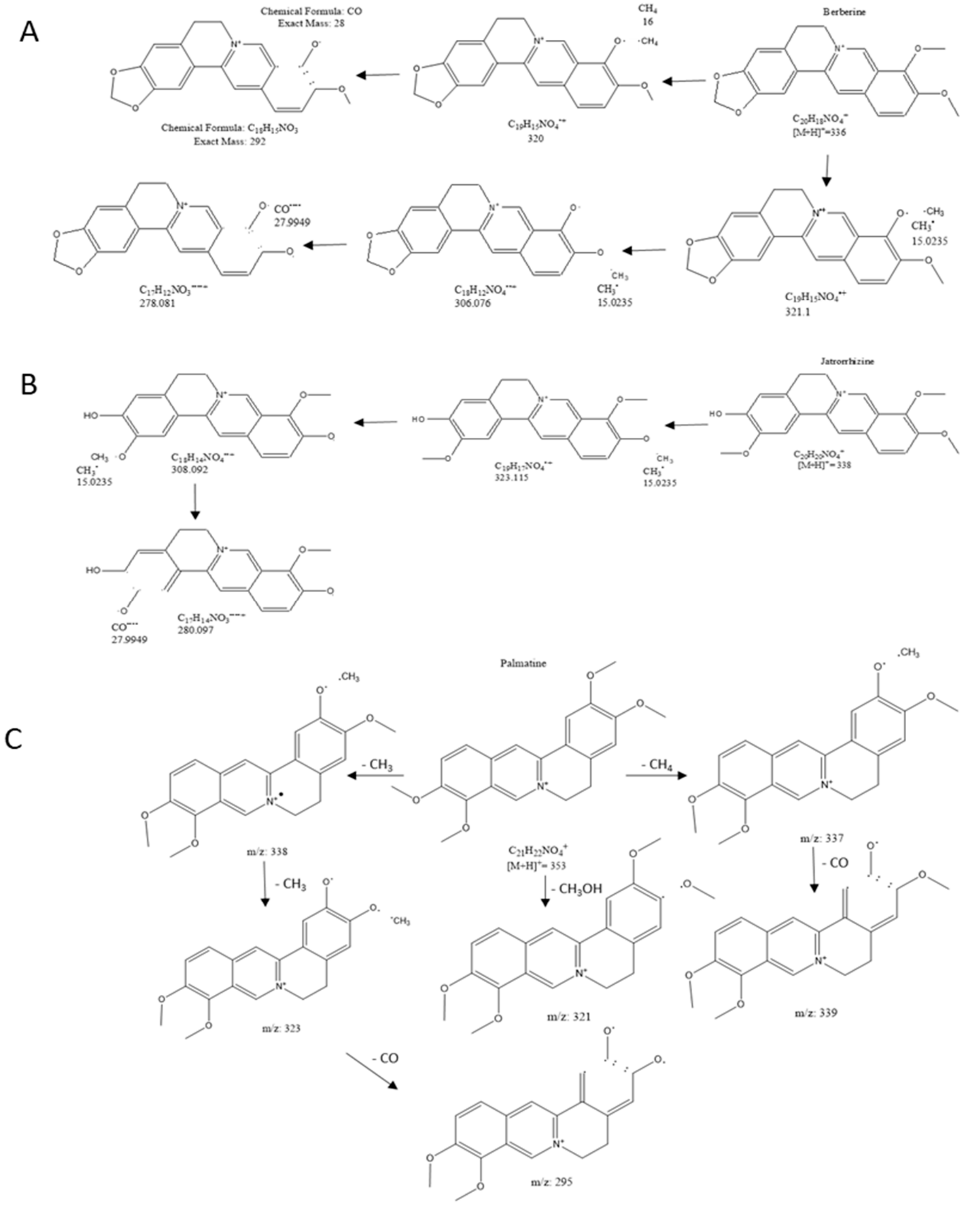

Figure S10. Proposed MS/MS fragmentation pathways of the identified protoberberine alkaloids in active fraction Fr 7: (A) berberine, (B) jatrorrhizine, and (C) palmatine.
